# Cross-cultural adaptation and validation of the Livello di Preparazione Alla Carriera Libero Professionale in Brazil

**DOI:** 10.1590/1518-8345.7849.4647

**Published:** 2025-08-18

**Authors:** Matheus Moraes Silva, Caroline Teodoro, Jouhanna do Carmo Menegaz, Glenda Roberta Oliveira Naiff Ferreira, Letícia de Lima Trindade, Alisson Fernandes Bolina, Queuam Ferreira Silva de Oliveira, Olga Maria Pimenta Lopes Ribeiro, Alessandro Stievano

**Affiliations:** 1 Universidade Federal do Pará, Faculdade de Enfermagem, Belém, PA, Brazil.; 2 Scholarship holder at the Coordenação de Aperfeiçoamento de Pessoal de Nível Superior (CAPES), Brazil.; 3 Universidade do Estado de Santa Catarina, Departamento de Enfermagem, Chapecó, SC, Brazil.; 4 Universidade Comunitária da Região de Chapecó, Chapecó, SC, Brazil.; 5 Universidade de Brasília, Faculdade de Ciências da Saúde, Brasília, DF, Brazil.; 6 Universidade Federal da Bahia, Salvador, BA, Brazil.; 7 Universidade do Estado da Bahia, Senhor do Bonfim, BA, Brazil.; 8 Escola Superior de Enfermagem do Porto, Porto, Portugal.; 9 University of Messina, Messina, Italy.

**Keywords:** Entrepreneurship, Students, Nursing, Job Market, Nursing, Private Duty, Factor Analysis, Statistical, Validation Study

## Abstract

to translate, adapt, and evaluate the psychometric properties of Livello di Preparazione Alla Carriera Libero Professionale inventory for use among final-year undergraduate nursing students in Brazil.

this is a cross-sectional methodological study conducted in two phases. In the first phase, the inventory was cross-culturally adapted to the Brazilian context. In the second phase, its psychometric properties were evaluated with 328 final-year undergraduate nursing students from different regions of Brazil.

confirmatory factor analysis confirmed a two-factor structure, consistent with the original Italian version. Factor loadings were significant (p < 0.001), ranging from 0.417 to 0.976. The adjustment indexes met the recommended criteria, validating the factor structure in the Brazilian context. Cronbach’s alpha and McDonald’s omega coefficients were greater than 0.70 for most factors and for the overall scale, demonstrating reliability.

the Brazilian version of the inventory demonstrated validity and reliability in assessing the preparation of final-year undergraduate nursing students for a liberal career, and it is also a useful tool for teachers and researchers to identify training gaps related to business entrepreneurship in this public.

## Highlights

**(1)** The inventory presents adequate construct validity and internal consistency. **(2)** It assesses knowledge about business entrepreneurship in nursing. **(3)** Subscale 1: assesses important factors for pricing services. **(4)** Subscale 2: assesses knowledge about the liberal career. **(5)** Validated with final-year nursing students.

## Introduction

The entrepreneurial nurse is defined by the International Council of Nurses (ICN) as someone who owns their own business, through which they offer nursing services, such as direct care, education, research, administration and/or consulting. In addition, they are directly responsible for the clients they have and may provide their services through direct agreements or subcontracts with public or private organizations^(1)^.

In this study, the independent professional is understood according to the Federal Council of Nursing (COFEN)^(2)^, which characterizes them as someone with a higher or technical education who practices their profession independently and is civilly responsible for any technical failures. The nurse, as an independent professional, has the freedom to practice his/her profession guaranteed by the Federal Constitution and by Law No. 7,498, of 1986^(3)^.

There are different types of entrepreneurship in the area, but only business entrepreneurship offers nurses opportunities for self-employment through innovative approaches in the creation and management of their own enterprise^(4)^. Business entrepreneurship in nursing also contributes to the development of new job opportunities and job creation, as well as to alignment with Sustainable Development Goal (SDG) 8, which aims to promote inclusive economic growth and decent work for all^(5)^.

However, despite the growing interest in entrepreneurship in nursing, entrepreneurial education is not yet a consolidated reality for undergraduate nursing students worldwide, nor does it follow a standard content^(6)^. Many training programs do not provide the necessary support to develop business management, finance, marketing, innovation and strategic leadership skills, which can limit the potential of nurses to start their own businesses^(7-8)^.

In Italy, for example, a survey of nursing students showed that, despite the growth of self-employment opportunities, there is a lack of structured programs that adequately prepare future nurses for independent careers^(9)^. In Brazil, the situation is similar. Although undergraduate nursing students recognize the importance of entrepreneurship, its approach during undergraduate studies is insufficient, with few opportunities for developing skills in this area^(10)^.

In line with these findings, a study conducted at a Brazilian public university revealed that 62.2% of nursing students reported that entrepreneurship is little explored during undergraduate studies^(11)^. Another study, which analyzed 130 undergraduate nursing courses at public higher education institutions in Brazil, showed that only 10.8% included a specific subject on entrepreneurship in their curriculum and/or pedagogical project; and, when included, the majority (78.6%) offered exclusively theoretical content, without the practical approach necessary for developing skills^(12)^.

Another study conducted in the Northeast region of Brazil revealed that, among the 412 Higher Education Institutions (HEIs) that offer undergraduate nursing courses, only 37 provided complete curricular documents, in which 17 subjects related to entrepreneurship and/or business were identified. The analysis of the pedagogical projects, course plans and syllabi identified that most of these subjects were theoretical (94.1%), while only 5.9% included a practical approach^(13)^. These data reinforce that the inclusion of the topic in undergraduate nursing curricula is still incipient in Brazil.

Given the gap in entrepreneurial training in this context, there is a lack of instruments to assess the preparation of undergraduate nursing students in relation to business entrepreneurship, considering the specificities of this area. To meet this need and evaluate educational strategies, a group of researchers in Italy developed and validated the inventory *Livello di Preparazione Alla Carriera Libero Professionale* (LPACIP) with the aim of assessing the preparedness of undergraduate nursing students to work in autonomous and independent careers. The LPACIP, validated in Italy with final-year undergraduate nursing students, demonstrated good psychometric properties, with adequate indices of construct validity and internal consistency^(14)^. Up until the time of this study, no cross-cultural adaptations or validations of the inventory in other countries had been identified in the literature.

Given the relevance of the LPACIP and the potential impact on entrepreneurial education in Brazilian nursing, which in recent years has signaled an increase in interest in business entrepreneurship, this study aimed to translate, adapt and evaluate the psychometric properties of the *Livello di Preparazione Alla Carriera Libero Professionale* inventory for use among final-year undergraduate nursing students in Brazil.

## Method

### Study design

This is a methodological, cross-sectional study developed in two phases. In the first, the process of cross-cultural adaptation was used to translate and adapt the LPACIP to the Brazilian context^(12)^. In the second phase, the translated and adapted inventory was subjected to tests to evaluate its psychometric properties, with emphasis on construct validity and internal consistency. The study was written in accordance with the STROBE (Strengthening the Reporting of Observational Studies in Epidemiology) guideline.

### Instrument

The LPACIP is composed of two subscales^(14)^. The first aims to identify the important factors for pricing independent nursing services and contains ten items distributed in two domains: *complessità dell’assistenza* (items one to seven) and *caratteristiche logistiche* (items eight to ten). The items are evaluated on a five-point ordinal scale, in which one indicates “not at all important” and five, “very important”. The second subscale addresses knowledge of the characteristics related to independent nursing work, composed of 24 items divided into two domains: *conoscenza delle regole amministrative* (items one to 12) and *conoscenza delle questioni relative a pensioni e previdenza* (items 13 to 24). Each item is evaluated on a five-point ordinal scale, in which one indicates “uninformed” and five, “fully informed”.

### Translation and cultural adaptation process

Following the guidelines for cross-cultural adaptation^(15)^, the LPACIP was translated and adapted to the Brazilian context through the following steps: direct translation, synthesis of translations, back-translation, review by a committee of experts, pre-testing, and approval of the adapted version by the author of the original instrument.

In the direct translation, two independent translators, both Brazilian and bilingual in Italian, translated the original instrument into Brazilian Portuguese, generating two versions (T1 and T2). Translator one had experience and knowledge of the concepts of the instrument, while translator two had no training in the health area and was not informed about the concepts. In the synthesis of the translations, T1 and T2 were carefully compared, and the translators and researchers developed the first consensual version (T1-2). Version T1-2 was back-translated into Italian independently (BT1 and BT2) by two bilingual translators in Portuguese, who had no training in the health area and were not familiar with the concepts of the inventory.

The expert committee was selected based on convenience, considering the members’ experience and availability to contribute to the cross-cultural adaptation of the instrument. The group was composed of two undergraduate nursing professors with experience in cross-cultural adaptation, an entrepreneurial nurse with five years of exclusive experience in the area, a final-year nursing student representing the target audience, and the four translators involved in the project.

The initial contact was made via email and, after agreeing to participate and signing the Free and Informed Consent Form (FICF) specific to this phase, the experts received the translated items to compare them with the original version. The committee was responsible for consolidating the pre-final version of the instrument. The evaluation of the translated version considered conceptual, cultural, semantic, and idiomatic equivalences. For this analysis, a four-point ordinal scale was used, in which one corresponded to “not equivalent” and four to “very equivalent”, allowing the validation of the content of each item. The scores assigned were used to calculate the Item Content Validity Index (I-CVI) and per subscale (S-CVI), as described in the ‘Data processing and analysis’ section. After this stage, an online meeting was held to discuss the discrepancies identified and approve the pre-final version of the inventory.

The pre-test was administered online between February and March 2024, using a questionnaire prepared in Google Forms^®^, structured in two parts. The first consisted of sociodemographic questions, and the second presented the pre-final version of the inventory approved by the expert committee. In addition, the understanding of the items was assessed through a space for comments at the end of each subscale, in which participants answered structured questions about the clarity of the language, difficulties in understanding, and whether their answers accurately reflected what they intended to express.

The inclusion criteria were: (1) being over 18 years old; (2) being in the final year of an undergraduate degree; and (3) regularly enrolled in a public or private HEI in an undergraduate nursing course. Participants were selected in a non-probabilistic manner and through voluntary participation, with no restrictions to pre-determined institutions. The survey was disseminated via *WhatsApp* ^®^ and *Instagram* ^®^, both on personal accounts and in research groups. In addition, the macro-project team assisted in distributing the survey to reach participants from different regions of Brazil. Data that did not meet the criteria were excluded.

The author of the original instrument monitored all stages of the cross-cultural adaptation, reviewing and approving the changes made, as well as the final version of the instrument.

### Participants, data collection and sample definition

In the second phase, to assess the psychometric properties, the same inclusion criteria as for the pre-test were applied. Furthermore, only participants who responded fully to the subscales were analyzed, since incomplete responses could compromise the validity of the instrument. Although there are methods for treating missing data in psychometrics, the literature indicates the lack of consensus on the ideal approach, and its practical application is still limited^(16)^. Therefore, incomplete responses and those that did not meet the established criteria were excluded.

The participants were defined to resemble the validation of the original inventory carried out in Italy, which also involved nursing students in the final year of their undergraduate studies^(14)^. Data collection took place online between April and September 2024, through a questionnaire prepared in Microsoft Forms^®^, whose dissemination followed the same strategies as the pre-test. The questionnaire was structured in two parts: the first collected sociodemographic data, such as gender, age, nationality, region of residence in Brazil, name of the HEI, and administrative category (public or private). The second part consisted of the final version of the inventory translated and adapted to the Brazilian context, called “*Preparação para Carreira Liberal*”, version for Brazilians (BV), also known as LPACIP-VB after the consensus of the experts.

The recommended sample size for Confirmatory Factor Analysis (CFA) is five to ten times the number of items in the instrument^(17)^. The original version of the LPACIP consisted of 34 items, but during the cross-cultural adaptation process, two items were excluded, resulting in a final version with 32 items in the LPACIP-VB. Thus, the estimated number of participants for this phase ranges from 160 to 320. A total of 361 responses were obtained, of which 33 were excluded because they did not meet the inclusion criteria. Thus, the final sample consisted of 328 participants.

### Data processing and analysis

The data were organized in Microsoft Excel^®^ spreadsheets and analyzed using the statistical software Jasp (version 0.19.1; Amsterdam, Netherlands). Descriptive statistics were used to characterize the sample. Quantitative variables were analyzed using mean, median, Standard Deviation (SD), minimum (Min.) and maximum (Max.), while nominal variables were analyzed using absolute (n) and relative (%) frequencies.

The scores assigned by the expert committee were used to calculate the I-CVI and S-CVI. The I-CVI represents the proportion of experts who assigned scores of three or four to each item, while the S-CVI corresponds to the mean I-CVI of all items. According to the literature, I-CVI values ≥ 0.80 and S-CVI ≥ 0.90 are considered adequate to ensure content validity^(18)^. To assess construct validity, a CFA was performed for each subscale of the inventory, using the Diagonally Weighted Least Squares (DWLS) method to estimate the parameters.

The CFA was conducted with a two-factor structure, the same as that confirmed in the Italian version^(14)^, in order to verify its applicability in a Brazilian sample. Before the CFA, the adequacy of the sample was verified by the Kaiser-Meyer-Olkin (KMO) test and Bartlett’s Sphericity Test, adopting the criterion of KMO greater than 0.70 and statistical significance (p ≤ 0.05) in the Bartlett test^(19-20)^. The CFA was performed using the Latent Variable Analysis (LAVAAN) package^(21)^ and assessed the quality of the model fit using the following indices: chi-square/degrees of freedom ratio (χ²/df), Normalized Fit Index (NFI), Comparative Fit Index (CFI), Tucker-Lewis Index (TLI), Root Mean Square Error of Approximation (RMSEA), and Standardized Root Mean Square Residual (SRMR). In addition, the correlation coefficients between the latent factors of the model were analyzed.

The model fit was considered adequate when the following criteria were met: χ²/df less than 5.0^(19)^; NFI, CFI, and TLI indices above 0.90^(22)^; RMSEA lower than 0.10 and SRMR lower than 0.08 indicate acceptable fit^(22)^. The factor loadings (λ) of the items were considered adequate when higher than 0.30^(19,23)^.

In addition, the coefficient of determination (R²) was calculated, which measures the proportion of variance of the dependent variable explained by the independent variable. The R² value ranges from zero to one, with values closer to one indicating an excellent fit of the model. In this study, R² was used to assess the quality of the fit of the model in relation to the observed data^(24)^. Internal consistency was assessed by Cronbach’s alpha (α) and McDonald’s omega (ω) coefficients for each factor and for the general subscale, considering values higher than 0.70 as references for both tests^(25-26)^.

### Ethical aspects

The study was approved by the Research Ethics Committee under opinion No. 6,430,383. Before the start of the research, authorization was obtained from the author of the original instrument to translate, adapt and validate the inventory for the Brazilian context. All participants were informed about the objectives of the study and participated voluntarily, signing the informed consent form.

## Results

### First phase: translation and cultural adaptation

The content validity of the LPACIP-VB was assessed by eight experts. In the cross-cultural adaptation process, the first subscale maintained ten items, showing excellent content validity, with I-CVI ranging from 0.875 to 1.000 and S-CVI of 0.975. In the second subscale, most items obtained I-CVI higher than 0.875, except for items 13 and 14 of factor two, whose values were 0.250 and 0.375, respectively.

These items addressed specific regulations of the Italian social security system, with no applicability in Brazil (Item 13: Functions of the Board of Directors of the National Institute of Social Security and Assistance to the Nursing Profession; Item 14: Functions of the General Advisory Board of the National Institute of Social Security and Assistance to the Nursing Profession). At the final consensus meeting, after discussion by the expert committee, it was decided to exclude these items to ensure that factor two accurately reflected Brazilian social security regulations and practices. With this exclusion, the final S-CVI was 0.965. Thus, the second subscale, which in the Italian version has 24 items^(14)^, now has 22 in the Brazilian version. The translations and the final version of the LPACIP-VB are available on the international Open Science Framework (OSF.io) platform, under the identifier no. 10.17605/OSF.IO/2SXD8 (https://doi.org/10.17605/OSF.IO/2SXD8).

As a result of the exclusion of the items, the calculation of the score for factor two of the second subscale was adjusted. Each domain of both subscales should be calculated by standardizing the raw rates into a single score from zero to 100. Thus, for the first subscale, in the “complexity of care” domain, the calculation is: [(sum of items 1 to 7)/7]; for the “logistical characteristics” domain, the calculation is: [(sum of items 8 to 10)/3]. For the second subscale, the “knowledge of administrative issues” domain is calculated as: [(sum of items 1 to 12)/12] and the “knowledge of benefits and retirement issues” domain as: [(sum of items 13 to 22)/10]. Higher scores indicate better preparation of students for liberal careers in nursing^(14)^.

The 47 students who participated in the pre-test were between 20 and 59 years old, with an average of 24.91 (median = 24.00; SD = 6.08), and the majority were female (87.23%). The geographic distribution was as follows: 23.40% from the Northeast; 21.28% from the North; 21.28% from the Midwest; 21.28% from the South and 12.77% from the Southeast. All participants stated that they had not encountered any difficulties in understanding the items of the subscales.

### Second phase: confirmatory factor analysis and internal consistency

Table 1 presents the characteristics of the participants in the second phase of the study (n=328). The geographic distribution covered all five regions of Brazil, with a higher concentration in the North region (44.21%). Regarding the administrative category of the HEIs, the majority of students (62.80%) were enrolled in private institutions. In total, students in the final year of undergraduate nursing courses from 34 HEIs participated, with the majority (70.58%) classified as universities (Table 1).

**Table 1-:** Characteristics of participants in the second phase of the study. Belém, PA, Brazil, 2024

**Nursing students (n=328)**	**n** * **(%)** ^†^
Age (years)	Mean 27.50; median 24.00; SD ^‡^ 7.58, minimum 20, maximum 60
Gender	
Female	277 (84.46%)
Male	50 (15.24%)
Other	1 (0.30%)
Nationality	
Brazillian	327 (99.70%)
Other	1 (0.30%)
Regions in Brazil	
North	145 (44.21%)
Northeast	81 (24.70%)
Midwest	40 (12.19%)
Southeast	22 (6.71%)
South	40 (12.19%)
Administrative category of HEIs ^§^	
Private	206 (62.80%)
Public	122 (37.20%)
Academic organizationof HEIs ^§^ (n*=34)	
University	24 (70.58%)
University Center	5 (14.71%)
College	5 (14.71%)

*n = Absolute frequency; ^†^% = Percentage frequency; ^‡^SD = Standard deviation; ^§^HEIs = Higher Education Institutions

Below, the results of the CFA and internal consistency of subscales one and two of the inventory are presented, respectively.

### First subscale: factors for determining activity prices

The adequacy of the data for the CFA was confirmed by the KMO test of 0.864 and by the significant Bartlett test (χ² = 1516.778, df = 45, p < 0.001). The standardized factor loadings are shown in Table 2 and ranged from 0.596 (item three) to 0.920 (item nine), all significant (p < 0.001), confirming that the items present good correlations with their respective factors. The covariance between the latent factors revealed a correlation of 0.757 between factor one and factor two, with a 95% Confidence Interval (CI) ranging from 0.686 to 0.828, Standard Error (SE) of 0.036 and p < 0.001. The R² analysis showed values ranging from 0.355 (item three) to 0.847 (item nine), indicating that a significant proportion of the variance of the items was explained by the factors (Table 2).

The model presented good adjustment indices: χ²/gl = 2.0 (χ² = 70.871, gl = 34, p < 0.001); NFI = 0.979; CFI = 0.989; TLI = 0.986; RMSEA = 0.058 (90% CI = 0.039–0.076) and SRMR = 0.066. These results confirm that the factor structure was confirmed (Table 2). Consistency showed Cronbach›s α values of 0.765 and McDonald’s ω of 0.760 for factor one, while for factor two the values were α of 0.673 and ω of 0.686. In the general subscale, the α was 0.812 and the ω of 0.811, evidencing satisfactory reliability (Table 3).

**Table 2-:** Confirmatory factor analysis of the subscale: factors for determining activity prices. Belém, PA, Brazil, 2024

**Items**	**Factorial loads**	**Standard error**	**R** ^2^ *	**P-value**
Factor one: care complexity				
1 Degree of complexity of the service	0.649	0.043	0.422	0.001
2 Same service provided in several appointments	0.610	0.042	0.372	0.001
3 Several services in the same service	0.596	0.048	0.355	0.001
4 Consecutive service requests. different from the first, by the same customer	0.639	0.044	0.408	0.001
5 Care interventions planning	0.775	0.040	0.601	0.001
6 Urgent nature of the service	0.827	0.033	0.683	0.001
7 Provision of medical-hospital equipment, materials and supplies for regular use by the professional	0.668	0.054	0.447	0.001
Factor two: logistical characteristics				
8 Distance from the customer’s home	0.619	0.044	0.383	0.001
9 Service during the night and early morning hours	0.920	0.031	0.847	0.001
10 Service on holidays	0.763	0.038	0.583	0.001
χ²/gl ^†^ = 2.0				
CFI ^‡^ = 0.989				
TLI ^§^ = 0.986				
NFI ^||^ = 0.979				
RMSEA ^¶^ = 0.058 (IC 90%** = 0.039–0.076)				
SRMR ^††^ = 0.066				

*R^2^ = Coefficient of Determination; ^†^χ²/gl = Chi-square/degrees of freedom; ^‡^CFI = Comparative Fit Index; ^§^TLI = Tucker-Lewis Index; ^||^NFI = Normed Fit Index; ^¶^RMSEA = Root Mean Square Error of Approximation; **CI = 90% confidence interval; ^††^SRMR = Standardized Root Mean Square Residual

**Table 3-:** Analysis of the internal consistency of the subscale: factors for determining activity prices. Belém, PA, Brazil, 2024

**Factors**	**Items**	**CI 95%** *	
		**Cronbach’s alpha**	**McDonald’s omega**
Factor one: care complexity	1, 2, 3, 4, 5, 6, 7	0.765 (0.724–0.802)	0.760 (0.720–0.800)
Factor two: logistical characteristics	8, 9, 10	0.673 (0.605–0.731)	0.686 (0.629–0.742)
Total		0.812 (0.779–0.840)	0.811 (0.781–0.842)

*CI 95% = 95% confidence interval

### Second subscale: knowledge about characteristics related to the liberal career

The adequacy of the data for the CFA was confirmed by the KMO test of 0.904 and by the significant Bartlett test (χ² = 11800.135, df = 231, p < 0.001). All standardized factor loadings were significant (p < 0.001), ranging from 0.417 (item 16) to 0.976 (item 21), as shown in Table 4. The covariance between the factors was also satisfactory, with a value of 0.806 (SE: 0.022; 95% CI: 0.764–0.848; p < 0.001), reflecting good correlation between Factor One and Factor Two.

The R^2^ ranged from 0.174 (item 16) to 0.953 (item 21), showing that a significant proportion of the variance of the items was explained by the factors (Table 4). The model fit presented adequate values: χ²/gl = 3.5 (χ² = 729.788, gl = 208, p < 0.001); NFI = 0.998; CFI = 0.999; TLI = 0.998; RMSEA = 0.088 (90% CI = 0.081 to 0.095; p < 0.000) and SRMR = 0.053, confirming the factorial structure (Table 4). Internal consistency was also satisfactory. For factor one, Cronbach›s α was 0.941 and McDonald’s ω was 0.944. In factor two, α was 0.960 and ω was 0.964. In the general subscale, α was 0.965 and ω was 0.967, demonstrating excellent reliability (Table 5).

**Table 4-:** Confirmatory factor analysis of the subscale: knowledge about characteristics related to the liberal career. Belém, PA, Brazil, 2024

**Items**	**Factorial loads**	**Standard error**	**R** ^2^ *	***P* -value**
Factor one: knowledge of administrative issues				
1 Fair remuneration	0.739	0.030	0.546	0.001
2 Health advertising	0.777	0.026	0.603	0.001
3 Ways in which the independent professional operates (clinic, office or nursing company)	0.449	0.045	0.201	0.001
4 Opening a clinic, office or nursing company	0.776	0.024	0.603	0.001
5 Management of client documentation at home and/or in the clinic, office or nursing company	0.933	0.010	0.871	0.001
6 Management of administrative documentation for the clinic, office or nursing company	0.940	0.011	0.883	0.001
7 Patient privacy (General Data Protection Law)	0.694	0.031	0.481	0.001
8 Regulatory obligations on budget preparation and contracting	0.901	0.012	0.812	0.001
9 Different types of tax regimes (simple national, presumed profit, real profit,...)	0.972	0.007	0.944	0.001
10 Different types of invoices (electronic invoice, electronic service invoice, single invoice, ...)	0.942	0.010	0.887	0.001
11 Liability insurance and accident policy	0.958	0.009	0.918	0.001
12 Role of the Nursing Council in relation to the liberal professional	0.808	0.019	0.653	0.001
Factor two: knowledge of benefits and retirement issues				
13 Functions of the National Social Security Institute (INSS)	0.934	0.007	0.873	0.001
14 Types of social security contributions from the National Institute of Social Security (INSS) for self-employed professionals (Normal Plan and Simplified Plan)	0.963	0.005	0.927	0.001
15 Differences between social security contributions from the National Institute of Social Security (INSS)	0.958	0.005	0.917	0.001
16 Differences between social security contributions from the National Institute of Social Security (INSS) for self-employed professionals (contribution based on the minimum wage or based on individual income)	0.417	0.036	0.174	0.001
17 Composition of the contribution amount	0.950	0.006	0.903	0.001
18 Social security benefits granted by the National Social Security Institute (INSS) (normal plan: retirement based on age, disability and contribution time, sickness benefit, maternity pay, imprisonment benefit, survivor’s pension, certificate of contribution time)	0.933	0.007	0.871	0.001
19 Social security benefits granted by the National Institute of Social Security (INSS) (simplified plan: retirement by age, sickness benefit, survivor’s pension, disability retirement, maternity pay, imprisonment benefit)	0.754	0.024	0.568	0.001
20 Differences between social security contributions and tax taxation	0.974	0.003	0.948	0.001
21 Deductibility of social security contributions	0.976	0.003	0.953	0.001
22 Regularity of contribution	0.957	0.005	0.915	0.001
χ²/gl ^†^ = 3.5				
CFI ^‡^ = 0.999				
TLI ^§^ = 0.998				
NFI ^||^ = 0.998				
RMSEA ^¶^ = 0.088 (IC 90%** = 0.081–0.095)				
SRMR ^††^ = 0.053				

*R^2^ = Coefficient of Determination; ^†^χ²/gl = Chi-square/degrees of freedom; ^‡^CFI = Comparative Fit Index; ^§^TLI = Tucker-Lewis Index; ^||^NFI = Normed Fit Index; ^¶^RMSEA = Root Mean Square Error of Approximation; **CI = 90% confidence interval; ^††^SRMR = Standardized Root Mean Square Residual

**Table 5-:** Analysis of the internal consistency of the subscale: knowledge about the characteristics related to the liberal career. Belém, PA, Brazil, 2024

**Factors**	**Items**	**CI 95%** *	
		**Cronbach’s alpha**	**McDonald’s omega**
Factor one: knowledge of administrative issues	1, 2, 3, 4, 5, 6, 7, 8, 9, 10, 11, 12	0.941 (0.932–0.950)	0.944 (0.935–0.953)
Factor two: knowledge of benefits and retirement issues	13, 14, 15, 16, 17, 18, 19, 20, 21, 22	0.960 (0.954–0.966)	0.964 (0.959–0.970)
Total		0.965 (0.959–0.970)	0.967 (0.962–0.973)

*CI 95% = 95% confidence interval

## Discussion

The results of this study indicate that the LPACIP-VB is a valid and reliable instrument for assessing the preparedness of final-year nursing students for liberal arts careers. As the first instrument validated for this purpose in Brazil, the inventory maintained its original factor structure, presenting robust psychometric properties that confirm its construct validity and internal consistency.

In the process of cross-cultural adaptation, the first subscale maintained ten items, while the second, originally with 24 in the Italian version^(14)^, now has 22 in the Brazilian version, due to the exclusion of items 13 and 14 of factor two, which addressed Italian social security regulations that are not applicable in Brazil. The decision was made by the expert committee, and the S-CVI of the second subscale resulted in 0.965 after the exclusion of the items. Removing items does not necessarily create a gap in the instrument; on the contrary, removing culturally inappropriate items can increase its quality, reflecting more accurately the reality of the country. This adaptation allows the instrument to better capture local experience, which mitigates ambiguous interpretations that could compromise the validity of the results^(27-29)^.

The translation, adaptation, and validation of the LPACIP in Brazil were motivated by the lack of validated instruments to assess the preparation of nursing students for independent careers. Until this study was prepared, the LPACIP is the only instrument that specifically measures knowledge about business entrepreneurship in the area, standing out in the face of the lack of reliable measures that identify the essential elements for autonomous practice. Although it was tested only once in its original study^(14)^, this research represents its second application, allowing the evaluation of its psychometric properties in a new cultural context. Its consolidated theoretical structure and the need for an instrument adapted to the Brazilian reality justify its validation and application in the country.

As highlighted in the original study^(14)^, further research is needed to test the construct validity of the LPACIP and to verify whether the preparation elements are associated with success in independent nursing practice. Future studies should consider longitudinal analyses and applications in different samples to strengthen the evidence of validity and reliability, expanding its applicability in different contexts.

The CFA confirmed that the two subscales maintained a two-factor factor structure, consistent with the original Italian version^(14)^. The factor loadings of the first subscale ranged from 0.596 to 0.920, while those of the second subscale ranged from 0.417 to 0.976, all above the recommended minimum value of 0.30(19,23), reinforcing the adequacy of the proposed theoretical model. Fit indices are descriptive measures that assess how well a CFA model fits the data^(30)^.

In this study, the fit indices of the LPACIP-VB were close to or higher than those of the Italian version. In the first subscale, the χ²/gl was better in Brazil (2.0 vs. 3.2 in Italy), as was the RMSEA, which was lower (0.058 vs. 0.075 in Italy). Furthermore, the incremental indices CFI (0.989) and TLI (0.986) surpassed those of the Italian version (CFI = 0.936; TLI = 0.907). In the second subscale, the Brazilian version presented a worse fit than the Italian one. The χ²/gl was 3.5 in Brazil and 2.3 in Italy, and the RMSEA was higher in Brazil (0.088 vs. 0.058). However, the incremental indices were high in both versions, with CFI of 0.999 in Brazil vs. 0.957 in Italy, and TLI of 0.998 vs. 0.950, indicating good model fit. The SRMR was 0.053 in Brazil and 0.033 in Italy^(14)^. Despite these differences, all indices remained within or close to the established parameters^(19,22)^, ensuring the validity of the instrument in the Brazilian context.

Exploratory Factor Analysis (EFA) is widely used in the initial stages of instrument development, as its purpose is to explore the factor structure underlying the data, without imposing a previous structure^(31)^, which was already done in the original version of the inventory^(14)^. In this study, we chose CFA, since it is the most appropriate method to test and confirm whether the empirical structure observed reflects the theoretical construct of interest in the population analyzed^(32)^. Thus, CFA was used in the Brazilian version to assess the applicability and adequacy of the factor structure previously identified in the Italian version in a new cultural context. This approach helps to ensure the validity and reliability of the instrument in different languages and scenarios^(32)^.

Regarding internal consistency, considering that Cronbach’s alpha (α) can be influenced by the number of items in a factor^(33)^, this study adopted an approach that complements that used in the validation process of the original inventory^(14)^. In addition to α, we also calculated McDonald’s ω coefficient, which provides a more restrictive estimate of reliability when based on the factor model (restrictive approach), assuming that items may have different factor loadings and measure different aspects of the construct^(34-35)^. Although ω is generally less sensitive to the number of items compared to α, factors with few items may present lower internal consistency values^(26,36)^.

Internal consistency was adequate for most factors and for the general subscales, with α and ω values above 0.70, reinforcing the construct validity of the instrument. However, factor two of subscale one, which contains only three items, presented slightly lower values, but still close to the established reference value, possibly influenced by the number of items in the factor. Although the α coefficient is often used in preference to other measures, there is still no clear consensus in the literature regarding its interpretation^(34)^. Nevertheless, the α values obtained in our study were close to those of the original inventory^(14)^.

It is believed that the implications of our results go beyond the psychometric validation of the LPACIP-VB. By using this instrument, professors and researchers will have the opportunity to assess in a structured manner the level of preparation of Brazilian final-year undergraduate nursing students to work in a liberal manner, an aspect that is increasingly relevant given the expansion of the scope of nursing practice beyond traditional work environments^(37)^.

In addition, it can be used as a reference to guide the development of academic curricula that are more aligned with strengthening the general skills required for liberal nursing practice. For example, if the assessment reveals low scores in areas such as pricing or knowledge about the characteristics of the liberal career, targeted strategies can be implemented to strengthen these areas based on the identified difficulties.

This study has some limitations that should be considered. First, cross-sectional data collection limits the ability to test the stability of each scale over time. Second, although the sample was obtained from different regions of Brazil, it may not fully represent the diversity of nursing students in their final year. Furthermore, the findings reflect the characteristics of the sample and the context in which the instrument was applied. Changes in the profile of students, especially in socioeconomic and demographic aspects, as well as different data collection methods, may impact its validity and reliability. Therefore, further investigations are recommended to assess its applicability in other contexts and populations. Finally, the use of self-administered measures may have introduced the potential for response bias or social desirability effects^(38)^.

Despite these limitations, the study contributes to the literature on nursing entrepreneurship by providing the first instrument validated for the Brazilian context, capable of measuring the preparedness of final-year nursing students for liberal practice. Furthermore, it can be used in future research to explore this preparation in different regions of Brazil, contributing to the advancement of scientific knowledge on this topic.

## Conclusion

The study translated, adapted and confirmed the construct validity and internal consistency of the LPACIP-VB to assess the preparedness of final-year undergraduate nursing students in Brazil to work in autonomous and independent careers. Therefore, it can be inferred that the instrument is effective for professors and researchers to identify gaps in teaching about entrepreneurship in nursing for this audience, enabling the development of theoretical and practical strategies that are more aligned with the students’ real difficulties.

In addition, the LPACIP-VB encourages discussion about service pricing, administrative and social security issues, fundamental aspects for future nurses to be able to work autonomously and develop more structured businesses in the area – a knowledge that is still little explored in academic training. Finally, it is worth noting that the use of the inventory is free of charge but requires the authorization of the corresponding author.
