# Supplementary material for: Cross-cultural adaptation and validation of the Livello di Preparazione Alla Carriera Libero Professionale in Brazil
Source: Rev Lat Am Enfermagem. 2025 Aug 18;33:e4647. doi: 10.1590/1518-8345.7849.4647 (PMC12364460; doi:10.1590/1518-8345.7849.4647)
Supplement: Translation

# Translation: Adaptação transcultural e validação do inventário *Livello di Preparazione Alla Carriera Libero Professionale* no Brasil

**DOI:** 10.1590/1518-8345.7849.4648

**Authors:** Matheus Moraes Silva, Caroline Teodoro, Jouhanna do Carmo Menegaz, Glenda Roberta Oliveira Naiff Ferreira, Letícia de Lima Trindade, Alisson Fernandes Bolina, Queuam Ferreira Silva de Oliveira, Olga Maria Pimenta Lopes Ribeiro, Alessandro Stievano

**Affiliations:** 1 Universidade Federal do Pará, Faculdade de Enfermagem, Belém, PA, Brasil.; 2 Bolsista da Coordenação de Aperfeiçoamento de Pessoal de Nível Superior (CAPES), Brasil.; 3 Universidade do Estado de Santa Catarina, Departamento de Enfermagem, Chapecó, SC, Brasil.; 4 Universidade Comunitária da Região de Chapecó, Chapecó, SC, Brasil.; 5 Universidade de Brasília, Faculdade de Ciências da Saúde, Brasília, DF, Brasil.; 6 Universidade Federal da Bahia, Salvador, BA, Brasil.; 7 Universidade do Estado da Bahia, Senhor do Bonfim, BA, Brasil.; 8 Escola Superior de Enfermagem do Porto, Porto, Portugal.; 9 University of Messina, Messina, Itália.

**Keywords:** Empreendedorismo, Estudantes de Enfermagem, Mercado de Trabalho, Prática Privada de Enfermagem, Análise Fatorial, Estudo de Validação

## Abstract

traduzir, adaptar e avaliar as propriedades psicométricas do inventário Livello di Preparazione Alla Carriera Libero Professionale para uso entre estudantes do último ano da graduação em enfermagem no Brasil.

estudo metodológico, de corte transversal, conduzido em duas fases. Na primeira, realizou-se a adaptação transcultural do inventário para o contexto brasileiro. Na segunda, suas propriedades psicométricas foram avaliadas com 328 estudantes do último ano da graduação em enfermagem, provenientes de diferentes regiões do Brasil.

a análise fatorial confirmatória confirmou uma estrutura de dois fatores, consistente com a versão original italiana. As cargas fatoriais foram significativas (p < 0,001), variando de 0,417 a 0,976. Os índices de ajuste atenderam aos critérios recomendados, validando a estrutura fatorial no contexto brasileiro. Os coeficientes alfa de Cronbach e ômega de McDonald foram superiores a 0,70 na maioria dos fatores e na escala geral, demonstrando confiabilidade.

a versão brasileira do inventário demonstrou validade e confiabilidade para avaliar a preparação de estudantes de graduação em enfermagem do último ano para a carreira liberal, sendo uma ferramenta útil para professores e pesquisadores identificarem lacunas formativas relacionadas ao empreendedorismo de negócios nesse público.

## Destaques

**(1)** O inventário apresenta validade de construto e consistência interna adequadas. **(2)** Avalia o conhecimento sobre empreendedorismo de negócios na enfermagem. **(3)** Subescala 1: avalia fatores importantes para a precificação de serviços. **(4)** Subescala 2: avalia o conhecimento sobre a carreira liberal. **(5)** Validado com estudantes do último ano da graduação em enfermagem.

## Introdução

O enfermeiro empreendedor é definido pelo Conselho Internacional de Enfermagem (ICN), como aquele que possui um negócio próprio, pelo qual oferece serviços de enfermagem, tais como cuidados diretos, educação, pesquisa, administração e/ou consultoria. Além disso, ele é diretamente responsável pelos clientes que atende, podendo prestar seus serviços por meio de acordos diretos ou subcontratos com organizações públicas ou privadas^(1)^.

Neste estudo, o profissional liberal é compreendido conforme parecer do Conselho Federal de Enfermagem (COFEN)^(2)^, que o caracteriza como alguém com nível superior ou técnico que exerce sua profissão com independência e é civilmente responsável por eventuais falhas técnicas. O enfermeiro, como profissional liberal, tem o livre exercício assegurado pela Constituição Federal e pela Lei nº 7.498, de 1986^(3)^.

Há diferentes tipos de empreendedorismo na área, mas somente o empreendedorismo de negócios oferece aos enfermeiros oportunidades de autoemprego por intermédio de abordagens inovadoras na criação e gestão de seu próprio empreendimento^(4)^. O empreendedorismo de negócios na enfermagem também contribui para o desenvolvimento de novas oportunidades de trabalho e geração de empregos, bem como para o alinhamento com o Objetivo de Desenvolvimento Sustentável (ODS) 8, que visa promover o crescimento econômico inclusivo e o trabalho decente para todos^(5)^.

Entretanto, apesar do crescente interesse pelo empreendedorismo na enfermagem, a educação empreendedora ainda não é uma realidade consolidada para estudantes de graduação em enfermagem em todo o mundo, tampouco segue um padrão de conteúdo^(6)^. Muitos programas de formação não fornecem o suporte necessário para desenvolver habilidades de gestão de negócios, finanças, marketing, inovação e liderança estratégica, o que pode limitar o potencial dos enfermeiros para abrir seus próprios negócios^(7-8)^.

Na Itália, por exemplo, uma pesquisa com estudantes de enfermagem evidenciou que, apesar do crescimento das oportunidades de trabalho autônomo, há uma falta de programas estruturados que preparem adequadamente os futuros enfermeiros para carreiras independentes^(9)^. No Brasil, a situação é semelhante, embora os estudantes de graduação em enfermagem reconheçam a importância do empreendedorismo, sua abordagem durante a graduação é insuficiente, com poucas oportunidades para o desenvolvimento de competências nessa área^(10)^.

Consoante a esses achados, o estudo realizado em uma universidade pública brasileira revelou que 62,2% dos estudantes de enfermagem relataram que o empreendedorismo é pouco explorado durante a graduação^(11)^. Outra investigação analisou 130 cursos de graduação em enfermagem de instituições públicas de ensino superior no Brasil, mostrando que apenas 10,8% incluíam na matriz curricular e/ou projeto pedagógico disciplina específica sobre empreendedorismo; e, quando incluída, a maioria (78,6%) oferecia conteúdo exclusivamente teórico, sem a abordagem prática necessária para o desenvolvimento de competências^(12)^.

O estudo realizado na região Nordeste do Brasil revelou que, entre as 412 Instituições de Ensino Superior (IES) que oferecem o curso de graduação em enfermagem, apenas 37 disponibilizaram documentos curriculares completos, nos quais foram identificadas 17 disciplinas relacionadas ao empreendedorismo e/ou empreendimento. A análise dos projetos pedagógicos, planos de curso e ementas identificou que a maioria dessas disciplinas era teórica (94,1%), enquanto apenas 5,9% incluía abordagem prática^(13)^. Esses dados reforçam que a inserção do tema nos currículos de graduação em enfermagem ainda é incipiente no Brasil.

Diante da lacuna na formação empreendedora nesse contexto, carece-se de instrumentos para avaliar o preparo dos estudantes de graduação em enfermagem em relação ao empreendedorismo de negócios, considerando as especificidades dessa área. Para suprir essa necessidade e avaliar estratégias educacionais, um grupo de pesquisadores na Itália desenvolveu e validou o inventário *Livello di Preparazione Alla Carriera Libero Professionale* (LPACIP) com o objetivo de avaliar o preparo dos estudantes de graduação em enfermagem para atuar em carreiras autônomas e independentes. O LPACIP, validado na Itália com estudantes do último ano da graduação em enfermagem, demonstrou boas propriedades psicométricas, com índices adequados de validade de construto e consistência interna^(14)^. Até o momento da elaboração deste estudo, não foram identificadas na literatura adaptações transculturais ou validações do inventário em outros países.

Considerando a relevância do LPACIP e o potencial impacto na educação empreendedora na enfermagem brasileira, nos últimos anos, sinaliza-se uma ampliação do interesse pelo empreendedorismo de negócios. Nesse sentido, este estudo teve como objetivo traduzir, adaptar e avaliar as propriedades psicométricas do inventário *Livello di Preparazione Alla Carriera Libero Professionale* para uso entre estudantes do último ano da graduação em enfermagem no Brasil.

## Método

### Delineamento do estudo

Trata-se de um estudo metodológico, de corte transversal, desenvolvido em duas fases. Na primeira, utilizou-se o processo de adaptação transcultural para traduzir e adaptar o LPACIP para o contexto brasileiro^(12)^. Na segunda, o inventário traduzido e adaptado foi submetido a testes para avaliar suas propriedades psicométricas, com ênfase na validade de construto e na consistência interna. O estudo foi redigido em conformidade com a diretriz STROBE (*Strengthening the Reporting of Observational Studies in Epidemiology*).

### Instrumento

O LPACIP é composto por duas subescalas^(14)^. A primeira visa identificar os fatores importantes para a precificação dos serviços liberais em enfermagem e contém dez itens distribuídos em dois domínios: *complessità dell’assistenza* (itens de um a sete) e *caratteristiche logistiche* (itens de oito a dez). Os itens são avaliados em uma escala ordinal de cinco pontos, na qual um indica “nada importante” e cinco, “muito importante”. A segunda subescala aborda o conhecimento das características relacionadas ao trabalho liberal em enfermagem, composta por 24 itens divididos em dois domínios: *conoscenza delle regole amministrative* (itens um a 12) e *conoscenza delle questioni relative a pensioni e previdenza* (itens 13 a 24). Cada item é avaliado em uma escala ordinal de cinco pontos, na qual um indica “desinformado” e cinco, “totalmente informado”.

### Processo de tradução e adaptação cultural

Seguindo as diretrizes de adaptação transcultural^(15)^, o LPACIP foi traduzido e adaptado para o contexto brasileiro por meio das seguintes etapas: tradução direta, síntese das traduções, retrotradução, revisão por comitê de especialistas, pré-teste e aprovação da versão adaptada pelo autor do instrumento original.

Na tradução direta, dois tradutores independentes, ambos brasileiros e bilíngues em italiano, traduziram o instrumento original para o português do Brasil gerando duas versões (T1 e T2). O tradutor um tinha experiência e conhecimento dos conceitos do instrumento, enquanto o tradutor dois não possuía formação na área da saúde e não foi informado sobre os conceitos. Na síntese das traduções, T1 e T2 foram cuidadosamente comparadas, e os tradutores e pesquisadores desenvolveram a primeira versão consensual (T1-2). A versão T1-2 foi retrotraduzida para o italiano de forma independente (RT1 e RT2) por dois tradutores bilíngues em português, que não possuíam formação na área da saúde e não estavam familiarizados com os conceitos do inventário.

O comitê de especialistas foi selecionado por conveniência, considerando a experiência e disponibilidade dos membros para contribuir com a adaptação transcultural do instrumento. O grupo foi composto por dois professores de graduação em enfermagem com experiência em adaptação transcultural, uma enfermeira empreendedora com cinco anos de atuação exclusiva na área, um estudante do último ano da graduação em enfermagem, representando o público-alvo, e os quatro tradutores envolvidos no projeto.

O contato inicial ocorreu por e-mail e, após aceitarem participar e assinarem o Termo de Consentimento Livre e Esclarecido (TCLE) específico para essa fase, os especialistas receberam os itens traduzidos para compará-los com a versão original. O comitê foi responsável por consolidar a versão pré-final do instrumento. A avaliação da versão traduzida considerou as equivalências conceitual, cultural, semântica e idiomática. Para essa análise, utilizou-se uma escala ordinal de quatro pontos, em que um correspondia a “não equivalente” e quatro a “muito equivalente”, permitindo a validação do conteúdo de cada item. As pontuações atribuídas foram utilizadas para calcular o Índice de Validade de Conteúdo por item (I-CVI) e por subescala (S-CVI), conforme descrito na seção ‘Tratamento e análise de dados’. Após essa etapa, realizou-se uma reunião online para discutir as discrepâncias identificadas e aprovar a versão pré-final do inventário.

O pré-teste foi aplicado online entre fevereiro e março de 2024, por meio de um questionário elaborado no *Google Forms* ^®^, estruturado em duas partes. A primeira constituía-se de questões sociodemográficas, e a segunda apresentava a versão pré-final do inventário aprovada pelo comitê de especialistas. Além disso, a compreensão dos itens foi avaliada por meio de um espaço para comentários ao final de cada subescala, no qual os participantes responderam a perguntas estruturadas sobre a clareza da linguagem, dificuldades de entendimento e se suas respostas refletiam com precisão o que desejavam expressar.

Os critérios de inclusão foram: (1) idade superior a 18 anos; (2) estar cursando o último ano da graduação; e (3) estar regularmente matriculado em uma IES pública ou privada no curso de graduação em enfermagem. A seleção dos participantes ocorreu de forma não probabilística e por adesão voluntária, sem restrição a instituições pré-determinadas. A divulgação foi realizada via *WhatsApp*® e *Instagram*®, tanto em contas pessoais quanto em grupos de pesquisa. Além disso, a equipe do macroprojeto auxiliou na distribuição da pesquisa para alcançar participantes de diferentes regiões do Brasil. Dados que não atenderam aos critérios foram excluídos.

O autor do instrumento original acompanhou todas as etapas da adaptação transcultural, revisando e aprovando as alterações realizadas, assim como a versão final do instrumento.

### Participantes, coleta de dados e definição da amostra

Na segunda fase, para avaliar as propriedades psicométricas, aplicaram-se os mesmos critérios de inclusão do pré-teste. Além disso, foram analisados apenas os participantes que responderam integralmente às subescalas, pois respostas incompletas poderiam comprometer a validade do instrumento. Embora existam métodos para tratar dados ausentes em psicometria, a literatura indica a ausência de um consenso sobre a abordagem ideal, e sua aplicação prática ainda é limitada^(16)^. Assim, foram excluídas as respostas incompletas e que não atendiam aos critérios estabelecidos.

Os participantes foram definidos para se assemelhar à validação do inventário original realizado na Itália, que também envolveu estudantes de enfermagem no último ano da graduação^(14)^. A coleta de dados ocorreu online entre abril e setembro de 2024, por meio de um questionário elaborado no *Microsoft Forms*®, cuja divulgação seguiu as mesmas estratégias do pré-teste. O questionário foi estruturado em duas partes: a primeira coletou dados sociodemográficos, como gênero, idade, nacionalidade, região de residência no Brasil, nome da IES e categoria administrativa (pública ou privada). A segunda parte consistiu na versão final do inventário traduzido e adaptado ao contexto brasileiro, denominado “Preparação para Carreira Liberal”, Versão Brasil (VB), também conhecido como LPACIP-VB, após o consenso dos especialistas.

O tamanho de amostra recomendado para Análise Fatorial Confirmatória (AFC) é de cinco a dez vezes o número de itens do instrumento^(17)^. A versão original do LPACIP constituía-se de 34 itens, mas, durante o processo de adaptação transcultural, dois itens foram excluídos, resultando em uma versão final com 32 itens no LPACIP-VB. Assim, o número estimado de participantes para esta fase varia de 160 a 320. Foram obtidas 361 respostas, das quais 33 foram excluídas por não atenderem aos critérios de inclusão. Assim, a amostra final foi constituída por 328 participantes.

### Tratamento e análise de dados

Os dados foram organizados em planilhas do *Microsoft Excel*® e analisados no *software* estatístico Jasp (versão 0.19.1; Amsterdã, Holanda). Estatísticas descritivas foram utilizadas para caracterizar a amostra. As variáveis quantitativas foram analisadas por meio de média, mediana, Desvio-Padrão (DP), mínimo (Min.) e máximo (Max.), enquanto as variáveis nominais por frequências absolutas (n) e relativas (%).

As pontuações atribuídas pelo comitê de especialistas foram utilizadas para calcular o I-CVI e S-CVI. O I-CVI representa a proporção de especialistas que atribuíram notas três ou quatro a cada item, enquanto o S-CVI corresponde à média do I-CVI de todos os itens. De acordo com a literatura, valores de I-CVI ≥ 0,80 e S-CVI ≥ 0,90 são considerados adequados para garantir a validade de conteúdo^(18)^.

Para avaliar a validade de construto, foi realizada uma AFC para cada subescala do inventário, utilizando o método de mínimos quadrados ponderados diagonalmente (*Diagonally Weighted Least Squares* – DWLS) para estimação dos parâmetros. A AFC foi conduzida com uma estrutura de dois fatores, a mesma confirmada na versão italiana^(14)^, a fim de verificar sua aplicabilidade em uma amostra brasileira. Antes da AFC, a adequação da amostra foi verificada pelo teste de Kaiser-Meyer-Olkin (KMO) e pelo Teste de Esfericidade de Bartlett, adotando-se o critério de KMO superior a 0,70 e significância estatística (p ≤ 0,05) no teste Bartlett^(19-20)^.

A AFC foi realizada com o pacote *Latent Variable Analysis* (LAVAAN)^(21)^ e avaliou a qualidade do ajuste do modelo por meio dos seguintes índices: razão qui-quadrado/graus de liberdade (χ²/gl), Índice de Ajuste Normalizado (*Normed Fit Index* – NFI), Índice de Ajuste Comparativo (*Comparative Fit Index* – CFI), Índice de Tucker-Lewis (TLI), Raiz do Erro Quadrático Médio de Aproximação (*Root Mean Square Error of Approximation* – RMSEA) e Raiz do Erro Quadrático Médio Residual (*Standardized Root Mean Square Residual* – SRMR). Além disso, foram analisados os coeficientes de correlação entre os fatores latentes do modelo.

O ajuste do modelo foi considerado adequado quando atendidos os seguintes critérios: χ²/gl inferior a 5,0^(19)^; índices NFI, CFI e TLI acima de 0,90^(22)^; RMSEA inferior a 0,10 e SRMR menor que 0,08 indicam ajustamento aceitável^(22)^. As cargas fatoriais (λ) dos itens foram consideradas adequadas quando superiores a 0,30^(19,23)^.

Adicionalmente, calculou-se o coeficiente de determinação (R²), o qual mede a proporção da variância da variável dependente explicada pela variável independente. O valor de R² varia entre zero e um, sendo que valores mais próximos de um indicam um excelente ajuste do modelo. Neste estudo, o R² foi utilizado para avaliar a qualidade do ajuste do modelo em relação aos dados observados^(24)^. A consistência interna foi avaliada pelos coeficientes alfa de Cronbach (α) e ômega de McDonald (ω) para cada fator e para a subescala geral, considerando valores superiores a 0,70 como referências para ambos os testes^(25-26)^.

### Aspectos éticos

O estudo foi aprovado pelo Comitê de Ética e Pesquisa sob o parecer nº 6.430.383. Antes do início da pesquisa, foi obtida a autorização do autor do instrumento original para traduzir, adaptar e validar o inventário para o contexto brasileiro. Todos os participantes foram informados sobre os objetivos do estudo e participaram de forma voluntária, assinando o TCLE.

## Resultados

### Primeira fase: tradução e adaptação cultural

A validade de conteúdo do LPACIP-VB foi avaliada pelos oito especialistas. No processo de adaptação transcultural, a primeira subescala manteve dez itens, apresentando excelente validade de conteúdo, com I-CVI variando de 0,875 a 1,000 e S-CVI de 0,975. Na segunda subescala**,** a maioria dos itens obteve I-CVI superior a 0,875, exceto os itens 13 e 14 do fator dois, cujos valores foram 0,250 e 0,375, respectivamente.

Esses itens abordavam regulamentações específicas do sistema previdenciário italiano, sem aplicabilidade no Brasil (Item 13: Funções do Conselho de Administração do Instituto Nacional de Previdência Social e Assistência à Profissão de Enfermagem; Item 14: Funções do Conselho Consultivo Geral do Instituto Nacional de Previdência Social e Assistência à Profissão de Enfermagem). Na reunião final de consenso, após discussão do comitê de especialistas, decidiu-se pela exclusão desses itens para garantir que o fator dois refletisse com precisão as regulamentações e práticas previdenciárias brasileiras. Com essa exclusão, o S-CVI final foi de 0,965. Assim, a segunda subescala, que na versão italiana possui 24 itens^(14)^, passou a ter 22 na versão brasileira. As traduções e a versão final do LPACIP-VB estão disponíveis na plataforma internacional *Open Science Framework* (OSF.io), sob o identificador nº 10.17605/OSF.IO/2SXD8 (https://doi.org/10.17605/OSF.IO/2SXD8).

Como resultado da exclusão dos itens, o cálculo da pontuação para o fator dois da segunda subescala foi ajustado. Cada domínio de ambas as subescalas deve ser calculado padronizando as taxas brutas em uma única pontuação de zero a 100. Assim, para a primeira subescala, no domínio “complexidade assistencial”, o cálculo é: [(soma dos itens 1 a 7)/7]; para o domínio “características logísticas”, o cálculo é: [(soma dos itens 8 a 10)/3]. Para a segunda subescala, o domínio “conhecimento de questões administrativas” é calculado como: [(soma dos itens 1 a 12)/12] e o domínio “conhecimento sobre questões de benefícios e aposentadoria” como: [(soma dos itens 13 a 22) /10]. Pontuações mais altas indicam melhor preparação dos estudantes para carreiras liberais na enfermagem^(14)^.

Os 47 estudantes que participaram do pré-teste tinham idades entre 20 e 59 anos, com média de 24,91 (mediana = 24,00; DP = 6,08), sendo a maioria do sexo feminino (87,23%). A distribuição geográfica foi a seguinte: 23,40% do Nordeste; 21,28% do Norte; 21,28% do Centro-Oeste; 21,28% do Sul e 12,77% do Sudeste. Todos os participantes afirmaram não ter encontrado dificuldades na compreensão dos itens das subescalas.

### Segunda fase: análise fatorial confirmatória e consistência interna

A Tabela 1 apresenta as características dos participantes da segunda fase do estudo (n=328). A distribuição geográfica abrangeu todas as cinco regiões do Brasil, com maior concentração na região Norte (44,21%). Em relação à categoria administrativa das IES, a maioria dos estudantes (62,80%) estava matriculada em privadas. No total, participaram estudantes do último ano de graduação em enfermagem de 34 IES, sendo a maioria (70,58%) classificada como universidade (Tabela 1).

**Tabela 1-:** Características dos participantes da segunda fase do estudo. Belém, PA, Brasil, 2024

**Estudantes de enfermagem (n=328)**	**n** * **(%)** ^†^
Idade (anos)	Média 27,50; mediana 24,00; DP ^‡^ 7,58, mínimo 20, máximo 60
Gênero	
Feminino	277 (84,46%)
Masculino	50 (15,24%)
Outro	1 (0,30%)
Nacionalidade	
Brasileira	327 (99,70%)
Outra	1 (0,30%)
Região do Brasil	
Norte	145 (44,21%)
Nordeste	81 (24,70%)
Centro-Oeste	40 (12,19%)
Sudeste	22 (6,71%)
Sul	40 (12,19%)
Categoria administrativa das IES ^§^	
Privada	206 (62,80%)
Pública	122 (37,20%)
Organização acadêmica das IES ^§^ (n*=34)	
Universidade	24 (70,58%)
Centro Universitário	5 (14,71%)
Faculdade	5 (14,71%)

*n = Frequência absoluta; ^†^% = Frequência percentual; ^‡^DP = Desvio-padrão; ^§^IES = Instituições de Ensino Superior

A seguir, são apresentados, respectivamente, os resultados da AFC e da consistência interna das subescalas um e dois do inventário.

### Primeira subescala: fatores para determinar os preços das atividades

A adequação dos dados para a AFC foi confirmada pelo teste KMO de 0,864 e pelo teste de Bartlett significativo (χ² = 1516,778, gl = 45, *p* < 0,001). As cargas fatoriais padronizadas são mostradas na Tabela 2 e variaram de 0,596 (item três) a 0,920 (item nove), todas significativas (*p* < 0,001), confirmando que os itens apresentam boas correlações com seus respectivos fatores. A covariância entre os fatores latentes revelou uma correlação de 0,757 entre o fator um e o fator dois, com um Intervalo de Confiança (IC) de 95% variando entre 0,686 e 0,828, Erro-Padrão (EP) de 0,036 e *p* < 0,001. A análise do R² mostrou valores variando de 0,355 (item três) a 0,847 (item nove), indicando que uma proporção significativa da variância dos itens foi explicada pelos fatores (Tabela 2).

O modelo apresentou bons índices de ajuste: χ²/gl = 2,0 (χ² = 70,871, gl = 34, *p* < 0,001); NFI = 0,979; CFI = 0,989; TLI = 0,986; RMSEA = 0,058 (IC 90% = 0,039–0,076) e SRMR = 0,066. Esses resultados confirmam que a estrutura fatorial foi confirmada (Tabela 2). A consistência apresentou valores de α de Cronbach de 0,765 e ω de McDonald de 0,760 para o fator um, enquanto, para o fator dois, os valores foram α de 0,673 e ω de 0,686. Na subescala geral, o α foi de 0,812 e o ω de 0,811, evidenciando uma confiabilidade satisfatória (Tabela 3).

**Tabela 2-:** Análise fatorial confirmatória da subescala: fatores para determinar os preços das atividades. Belém, PA, Brasil, 2024

**Itens**	**Cargas fatoriais**	**Erro-padrão**	**R** ^2^ *	***P* -valor**
Fator um: complexidade assistencial				
1 Grau de complexidade assistencial do serviço	0,649	0,043	0,422	0,001
2 Mesmo serviço prestado em vários atendimentos	0,610	0,042	0,372	0,001
3 Vários serviços no mesmo atendimento	0,596	0,048	0,355	0,001
4 Solicitações de serviços consecutivos, diferentes do primeiro, pelo mesmo cliente	0,639	0,044	0,408	0,001
5 Planejamento das intervenções assistenciais	0,775	0,040	0,601	0,001
6 Caráter de urgência do serviço	0,827	0,033	0,683	0,001
7 Fornecimento de equipamentos, materiais e insumos médico-hospitalares de uso habitual pelo profissional	0,668	0,054	0,447	0,001
Fator dois: características logísticas				
8 Distância do domicílio do cliente	0,619	0,044	0,383	0,001
9 Serviço nos períodos da noite e madrugada	0,920	0,031	0,847	0,001
10 Serviço em feriados	0,763	0,038	0,583	0,001
χ²/gl ^†^ = 2,0				
CFI ^‡^ = 0,989				
TLI ^§^ = 0,986				
NFI ^||^ = 0,979				
RMSEA ^¶^ = 0,058 (IC 90%** = 0,039–0,076)				
SRMR ^††^ = 0,066				

*R^2^ = Coeficiente de Determinação; ^†^χ²/gl = Qui-quadrado/graus de liberdade; ^‡^CFI = *Comparative Fit Index*; ^§^TLI = Tucker-Lewis *Index*; ^||^NFI = *Normed Fit Index*; ^¶^RMSEA = *Root Mean Square Error of Approximation*; **IC = Intervalo de Confiança de 90%; ^††^SRMR = *Standardized Root Mean Square Residual*

**Tabela 3-:** Análise da consistência interna da subescala: fatores para determinação dos preços das atividades. Belém, PA, Brasil, 2024

**Fatores**	**Itens**	**IC 95%** *	
		**Alfa de Cronbach**	**Ômega de McDonalds**
Fator um: Complexidade Assistencial	1, 2, 3, 4, 5, 6, 7	0,765 (0,724–0,802)	0,760 (0,720–0,800)
Fator dois: Características Logísticas	8, 9, 10	0,673 (0,605–0,731)	0,686 (0,629–0,742)
Total		0,812 (0,779–0,840)	0,811 (0,781–0,842)

*IC 95% = Intervalo de Confiança de 95%

### Segunda subescala: conhecimento sobre as características relacionadas à carreira liberal

A adequação dos dados para a AFC foi confirmada pelo teste KMO de 0,904 e pelo teste de Bartlett significativo (χ² = 11800,135, gl = 231, *p* < 0,001). Todas as cargas fatoriais padronizadas foram significativas (*p* < 0,001), variando de 0,417 (item 16) a 0,976 (item 21), conforme apresentado na Tabela 4. A covariância entre os fatores também foi satisfatória, com um valor de 0,806 (EP: 0,022; IC 95%: 0,764–0,848; *p* < 0,001), refletindo boa correlação entre o Fator um e Fator dois.

O R^2^ variou de 0,174 (item 16) a 0,953 (item 21), evidenciando que uma proporção significativa da variância dos itens foi explicada pelos fatores (Tabela 4). O ajuste do modelo apresentou os valores adequados: χ²/gl = 3,5 (χ² = 729,788, gl = 208, *p* < 0,001); NFI = 0,998; CFI = 0,999; TLI = 0,998; RMSEA = 0,088 (IC 90% = 0,081 a 0,095; *p* < 0,000) e SRMR = 0,053, confirmando a estrutura fatorial (Tabela 4). A consistência interna também foi satisfatória. Para o fator um, o α de Cronbach foi de 0,941 e o ω de McDonald de 0,944. No fator dois, α foi de 0,960 e ω de 0,964. Na subescala geral, o α foi de 0,965 e ω de 0,967, evidenciando excelente confiabilidade (Tabela 5).

**Tabela 4-:** Análise fatorial confirmatória da subescala: conhecimento sobre as características relacionadas à carreira liberal. Belém, PA, Brasil, 2024

**Itens**	Cargas fatoriais	**Erro-padrão**	**R** ^2^ *	***P* -valor**
Fator um: conhecimento sobre questões administrativas				
1 Remuneração justa	0,739	0,030	0,546	0,001
2 Propaganda em saúde	0,777	0,026	0,603	0,001
3 Formas de atuação do profissional liberal (clínica, consultório ou empresa de enfermagem)	0,449	0,045	0,201	0,001
4 Abertura de uma clínica, consultório ou empresa de enfermagem	0,776	0,024	0,603	0,001
5 Gestão da documentação do cliente em domicílio e/ou na clínica, consultório ou empresa de enfermagem	0,933	0,010	0,871	0,001
6 Gestão da documentação administrativa da clínica, consultório ou empresa de enfermagem	0,940	0,011	0,883	0,001
7 Privacidade do paciente (Lei Geral da Proteção de Dados Pessoais)	0,694	0,031	0,481	0,001
8 Obrigações regulatórias sobre elaboração de orçamento e contratualização	0,901	0,012	0,812	0,001
9 Diferentes tipos de regimes tributários (simples nacional, lucro presumido, lucro real, ...)	0,972	0,007	0,944	0,001
10 Diferentes tipos de notas (nota fiscal eletrônica, nota fiscal eletrônica de serviço, nota fiscal avulsa, ...)	0,942	0,010	0,887	0,001
11 Seguro de responsabilidade civil e apólice de acidentes	0,958	0,009	0,918	0,001
12 Papel do Conselho de Enfermagem em relação ao profissional liberal	0,808	0,019	0,653	0,001
Fator dois: conhecimento sobre questões de benefícios e aposentadoria				
13 Funções do Instituto Nacional do Seguro Social (INSS)	0,934	0,007	0,873	0,001
14 Tipos de contribuição previdenciárias do Instituto Nacional do Seguro Social (INSS) para profissionais liberais (Plano Normal e Plano Simplificado)	0,963	0,005	0,927	0,001
15 Diferenças entre contribuição previdenciárias do Instituto Nacional do Seguro Social (INSS)	0,958	0,005	0,917	0,001
16 Diferenças entre contribuição previdenciárias do Instituto Nacional do Seguro Social (INSS) para profissionais liberais (contribuição com base no salário-mínimo ou com base na renda individual)	0,417	0,036	0,174	0,001
17 Composição do montante da contribuição	0,950	0,006	0,903	0,001
18 Benefícios previdenciários concedidos pelo Instituto Nacional do Seguro Social (INSS) (plano normal: aposentadoria por idade, invalidez e tempo de contribuição, auxílio-doença, salário maternidade, auxílio reclusão, pensão por morte, certidão de tempo de contribuição)	0,933	0,007	0,871	0,001
19 Benefícios previdenciários concedidos pelo Instituto Nacional do Seguro Social (INSS) (plano simplificado: aposentadoria por idade, auxílio-doença, pensão por morte, aposentadoria por invalidez, salário maternidade, auxílio reclusão)	0,754	0,024	0,568	0,001
20 Diferenças entre as contribuições previdenciárias e a tributação fiscal	0,974	0,003	0,948	0,001
21 Dedutibilidade da contribuição previdenciária	0,976	0,003	0,953	0,001
22 Regularidade de contribuição	0,957	0,005	0,915	0,001
χ²/gl ^†^ = 3,5				
CFI ^‡^ = 0,999				
TLI ^§^ = 0,998				
NFI ^||^ = 0,998				
RMSEA ^¶^ = 0,088 (IC 90%** = 0,081–0,095)				
SRMR ^††^ = 0,053				

*R^2^ = Coeficiente de Determinação; ^†^χ²/gl = Qui-quadrado/graus de liberdade; ^‡^CFI = *Comparative Fit Index*; ^§^TLI = Tucker-Lewis *Index*; ^||^NFI = *Normed Fit Index*; ^¶^RMSEA = *Root Mean Square Error of Approximation*; **IC = Intervalo de Confiança de 90%; ^††^SRMR = *Standardized Root Mean Square Residual*

**Tabela 5-:** Análise da consistência interna da subescala: conhecimento sobre as características relacionadas a carreira liberal. Belém, PA, Brasil, 2024

**Fatores**	**Itens**	**IC 95%** *	
		**Alfa de Cronbach**	**Ômega de McDonalds**
Fator um: conhecimento sobre questões administrativas	1, 2, 3, 4, 5, 6, 7, 8, 9, 10, 11, 12	0,941 (0,932–0,950)	0,944 (0,935–0,953)
Fator dois: conhecimento sobre questões de benefícios e aposentadoria	13, 14, 15, 16, 17, 18, 19, 20, 21, 22	0,960 (0,954–0,966)	0,964 (0,959–0,970)
Total		0,965 (0,959–0,970)	0,967 (0,962–0,973)

*IC 95% = Intervalo de Confiança de 95%

## Discussão

Os resultados deste estudo indicam que o LPACIP-VB é um instrumento válido e confiável para avaliar a preparação de estudantes do último ano da graduação em enfermagem para carreiras liberais. Sendo o primeiro validado com esse propósito no Brasil, o inventário manteve sua estrutura fatorial original, apresentando propriedades psicométricas robustas que confirmam sua validade de construto e consistência interna.

No processo de adaptação transcultural, a primeira subescala manteve dez itens, enquanto a segunda, originalmente com 24 na versão italiana^(14)^, passou a ter 22 na brasileira, devido à exclusão dos itens 13 e 14 do fator dois, que abordavam regulamentações previdenciárias italianas sem aplicabilidade no Brasil. A decisão foi tomada pelo comitê de especialistas, e o S-CVI da segunda subescala resultou em 0,965 após a exclusão dos itens. A remoção de itens não ocasiona necessariamente uma lacuna no instrumento; ao contrário, remover itens culturalmente inadequados pode aumentar sua qualidade, refletindo de forma mais precisa a realidade do país. Essa adaptação permite que o instrumento capture melhor a experiência local, o que mitiga interpretações ambíguas que poderiam comprometer a validade dos resultados^(27-29)^.

A tradução, adaptação e validação do LPACIP no Brasil foram motivadas pela ausência de instrumentos validados para avaliar a preparação de estudantes de enfermagem para carreiras liberais. Até a elaboração deste estudo, o LPACIP é o único instrumento que mede especificamente o conhecimento acerca do empreendedorismo de negócios na área, destacando-se diante da carência de medidas confiáveis que identifiquem os elementos essenciais para a prática autônoma. Embora tenha sido testado apenas uma vez em seu estudo original^(14)^, esta pesquisa representa sua segunda aplicação, permitindo avaliar suas propriedades psicométricas em um novo contexto cultural. Sua estrutura teórica consolidada e a necessidade de um instrumento adaptado à realidade brasileira justificam sua validação e aplicação no país.

Conforme ressaltado no estudo original^(14)^, mais investigações são necessárias para testar a validade de construto do LPACIP e verificar se os elementos de preparação se associam ao sucesso na prática liberal da enfermagem. Estudos futuros devem considerar análises longitudinais e aplicações em diferentes amostras para fortalecer as evidências de validade e confiabilidade, ampliando sua aplicabilidade em diversos contextos.

A AFC confirmou que as duas subescala mantiveram estrutura fatorial de dois fatores, consistente com a versão original italiana^(14)^. As cargas fatoriais da primeira subescala variaram de 0,596 a 0,920, enquanto as da segunda subescala ficaram entre 0,417 e 0,976, todas acima do valor mínimo recomendado de 0,30^(19,23)^, reforçando a adequação do modelo teórico proposto. Os índices de ajuste são medidas descritivas que avaliam o quão bem um modelo de AFC se ajusta aos dados^(30)^.

Neste estudo, os índices de ajuste do LPACIP-VB foram próximos ou superiores aos da versão italiana. Na primeira subescala, o χ²/gl foi melhor no Brasil (2,0 vs. 3,2 na Itália), assim como o RMSEA, que foi menor (0,058 vs. 0,075 na Itália). Além disso, os índices incrementais CFI (0,989) e TLI (0,986) superaram os da versão italiana (CFI = 0,936; TLI = 0,907). Na segunda subescala, a versão brasileira apresentou ajuste inferior à italiana. O χ²/gl foi de 3,5 no Brasil e 2,3 na Itália, e o RMSEA foi maior no Brasil (0,088 vs. 0,058). No entanto, os índices incrementais foram elevados em ambas as versões, com CFI de 0,999 no Brasil vs. 0,957 na Itália, e TLI de 0,998 vs. 0,950, indicando boa adequação do modelo. O SRMR foi 0,053 no Brasil e 0,033 na Itália^(14)^. Apesar dessas diferenças, todos os índices permaneceram dentro ou próximos dos parâmetros estabelecidos^(19,22)^, assegurando a validade do instrumento no contexto brasileiro.

A Análise Fatorial Exploratória (AFE) é amplamente utilizada nas etapas iniciais de desenvolvimento de instrumentos, pois seu propósito é explorar a estrutura fatorial subjacente aos dados, sem impor uma estrutura prévia^(31)^, o que já foi feito na versão original do inventário^(14)^. Neste estudo, optamos pela AFC, uma vez que é o método mais adequado para testar e confirmar se a estrutura empírica observada reflete o construto teórico de interesse na população analisada^(32)^. Dessa forma, a AFC foi empregada na versão brasileira para avaliar a aplicabilidade e a adequação da estrutura fatorial previamente identificada na versão italiana em um novo contexto cultural. Essa abordagem contribui para garantir a validade e a confiabilidade do instrumento em diferentes idiomas e cenários^(32)^.

Em relação à consistência interna, considerando que o alfa de Cronbach (α) pode ser influenciado pelo número de itens de um fator^(33)^, este estudo adotou uma abordagem complementar à utilizada no processo de validação do inventário original^(14)^. Além do α, também calculamos o coeficiente ω de McDonald, que oferece uma estimativa mais restritiva da confiabilidade ao se basear no modelo fatorial (abordagem restritiva), assumindo que os itens podem ter diferentes cargas fatoriais e medir diferentes aspectos do construto^(34-35)^. Embora o ω seja geralmente menos sensível ao número de itens em comparação ao α, fatores com poucos itens podem apresentar valores mais baixos de consistência interna^(26,36)^.

A consistência interna foi adequada para a maioria dos fatores e para as subescalas gerais, com valores de α e ω acima de 0,70, reforçando a validade de construto do instrumento. No entanto, o fator dois da subescala um, que contém apenas três itens, apresentou valores ligeiramente inferiores, mas ainda próximos ao valor de referência estabelecido, possivelmente influenciados pelo número de itens no fator. Embora o coeficiente α seja frequentemente utilizado em detrimento de outras medidas, ainda não há um consenso claro na literatura sobre sua interpretação^(34)^. Ainda assim, os valores de α obtidos em nosso estudo foram próximos aos do inventário original^(14)^.

Acredita-se que as implicações de nossos resultados vão além da validação psicométrica do LPACIP-VB. Ao utilizar este instrumento, professores e pesquisadores terão a oportunidade de avaliar de forma estruturada o nível de preparo dos estudantes do último ano de graduação em enfermagem brasileiros para atuar de forma liberal, um aspecto cada vez mais relevante diante da expansão do escopo de prática dos enfermeiros, para além dos ambientes tradicionais de trabalho^(37)^.

Além disso, pode ser utilizado como referência para orientar o desenvolvimento de currículos acadêmicos mais alinhados para o fortalecimento das habilidades gerais requeridas para a prática liberal na enfermagem. Por exemplo, se a avaliação revelar pontuações baixas em áreas como a determinação de preços ou o conhecimento sobre as características da carreira liberal, estratégias direcionadas podem ser implementadas para reforçar essas áreas com base nas dificuldades identificadas.

Este estudo apresenta algumas limitações que devem ser consideradas. Primeiro, a coleta de dados transversal limita a capacidade de testar a estabilidade de cada escala ao longo do tempo. Segundo, embora a amostra tenha sido obtida de diferentes regiões do Brasil, ela pode não representar completamente a diversidade dos estudantes de enfermagem cursando o último ano. Além disso, os achados refletem as características da amostra e o contexto de aplicação do instrumento. Mudanças no perfil dos estudantes, especialmente em aspectos socioeconômicos e demográficos, bem como diferentes métodos de coleta de dados, podem impactar sua validade e confiabilidade. Dessa forma, novas investigações são recomendadas para avaliar sua aplicabilidade em outros contextos e populações. Por fim, o uso de medidas autoadministradas pode ter introduzido o potencial para viés de resposta ou efeitos de desejabilidade social^(38)^.

Apesar dessas limitações, o estudo contribui para a literatura sobre o empreendedorismo em enfermagem ao fornecer o primeiro instrumento validado para o contexto brasileiro, capaz de medir a preparação de estudantes de enfermagem do último ano para a prática liberal. Além disso, pode ser usado em pesquisas futuras para explorar essa preparação em diferentes regiões do Brasil, contribuindo para o avanço do conhecimento científico acerca dessa temática.

## Conclusão

O estudo traduziu, adaptou e confirmou a validade de construto e a consistência interna do LPACIP–VB para avaliar a preparação dos estudantes de enfermagem do último ano da graduação no Brasil para atuar em carreiras autônomas e independentes. Infere-se, portanto, que o instrumento é eficaz para que professores e pesquisadores identifiquem lacunas no ensino sobre empreendedorismo em enfermagem nesse público, possibilitando o desenvolvimento de estratégias teóricas e práticas mais alinhadas às reais dificuldades dos estudantes.

Além disso, o LPACIP-VB fomenta a discussão sobre precificação de serviços, questões administrativas e previdenciárias, aspectos fundamentais para que os futuros enfermeiros possam atuar de forma liberal e desenvolver negócios mais estruturados na área – um conhecimento ainda pouco explorado na formação acadêmica. Por fim, ressalta-se que o uso do inventário é gratuito, mas requer a autorização do autor correspondente.

# Translation: Adaptación transcultural y validación del inventario *Livello di Preparazione Alla Carriera Libero Professionale* en Brasil

**DOI:** 10.1590/1518-8345.7849.4646

**Authors:** Matheus Moraes Silva, Caroline Teodoro, Jouhanna do Carmo Menegaz, Glenda Roberta Oliveira Naiff Ferreira, Letícia de Lima Trindade, Alisson Fernandes Bolina, Queuam Ferreira Silva de Oliveira, Olga Maria Pimenta Lopes Ribeiro, Alessandro Stievano

**Affiliations:** 1 Universidade Federal do Pará, Faculdade de Enfermagem, Belém, PA, Brasil.; 2 Becario de la Coordenação de Aperfeiçoamento de Pessoal de Nível Superior (CAPES), Brasil.; 3 Universidade do Estado de Santa Catarina, Departamento de Enfermagem, Chapecó, SC, Brasil.; 4 Universidade Comunitária da Região de Chapecó, Chapecó, SC, Brasil.; 5 Universidade de Brasília, Faculdade de Ciências da Saúde, Brasília, DF, Brasil.; 6 Universidade Federal da Bahia, Salvador, BA, Brasil.; 7 Universidade do Estado da Bahia, Senhor do Bonfim, BA, Brasil.; 8 Escola Superior de Enfermagem do Porto, Porto, Portugal.; 9 University of Messina, Messina, Italia.

**Keywords:** Emprendimiento, Estudiantes de Enfermería, Mercado de Trabajo, Práctica Privada de Enfermería, Análisis Factorial, Estudio de Validación

## Abstract

traducir, adaptar y evaluar las propiedades psicométricas del inventario *Livello di Preparazione Alla Carriera Libero Professionale* para su uso entre estudiantes del último año de la carrera de enfermería en Brasil.

estudio metodológico, de corte transversal, conducido en dos fases. En la primera, se realizó la adaptación transcultural del inventario al contexto brasileño. En la segunda, se evaluaron sus propiedades psicométricas con 328 estudiantes del último año de la carrera de enfermería, provenientes de diferentes regiones de Brasil.

el análisis factorial confirmatorio confirmó una estructura de dos factores, consistente con la versión original italiana. Las cargas factoriales fueron significativas (*p* < 0,001), variando de 0,417 a 0,976. Los índices de ajuste cumplieron con los criterios recomendados, validando la estructura factorial en el contexto brasileño. Los coeficientes alfa de Cronbach y omega de McDonald fueron superiores a 0,70 en la mayoría de los factores y en la escala general, demostrando confiabilidad.

la versión brasileña del inventario demostró validez y confiabilidad para evaluar la preparación de estudiantes de enfermería del último año para la carrera liberal, siendo una herramienta útil para que docentes e investigadores identifiquen lagunas formativas relacionadas con el emprendimiento de negocios en este público.

## Destacados

**(1)** El inventario presenta validez de constructo y consistencia interna adecuadas. **(2)** Evalúa el conocimiento sobre emprendimiento de negocios en enfermería. **(3)** Subescala 1: evalúa factores importantes para la fijación de precios de los servicios. **(4)** Subescala 2: evalúa el conocimiento sobre la carrera liberal. **(5)** Validado con estudiantes del último año de la carrera de enfermería.

## Introducción

El enfermero emprendedor es definido por el Consejo Internacional de Enfermería (ICN) como aquel que posee un negocio propio mediante el cual ofrece servicios de enfermería, tales como cuidados directos, educación, investigación, administración y/o consultoría. Además, es directamente responsable por los clientes que atiende, pudiendo prestar sus servicios por medio de acuerdos directos o subcontratos con organizaciones públicas o privadas^(1)^.

En este estudio, el profesional liberal se entiende según el dictamen del Consejo Federal de Enfermería (COFEN)^(2)^, que lo caracteriza como una persona con formación superior o técnica que ejerce su profesión con independencia y es civilmente responsable por eventuales fallos técnicos. El enfermero, como profesional liberal, tiene el libre ejercicio asegurado por la Constitución Federal y por la Ley n.º 7.498, de 1986^(3)^.

Existen diferentes tipos de emprendimiento en el área, pero solamente el emprendimiento de negocios ofrece a los enfermeros oportunidades de autoempleo mediante enfoques innovadores en la creación y gestión de su propio emprendimiento^(4)^. El emprendimiento de negocios en enfermería también contribuye al desarrollo de nuevas oportunidades laborales y generación de empleos, así como al alineamiento con el Objetivo de Desarrollo Sostenible (ODS) 8, que busca promover el crecimiento económico inclusivo y el trabajo decente para todos^(5)^.

Sin embargo, a pesar del creciente interés por el emprendimiento en enfermería, la educación emprendedora aún no es una realidad consolidada para los estudiantes de grado en enfermería a nivel mundial, ni sigue un estándar de contenido^(6)^. Muchos programas de formación no proporcionan el soporte necesario para desarrollar habilidades en gestión de negocios, finanzas, mercadotecnia, innovación y liderazgo estratégico, lo cual puede limitar el potencial de los enfermeros para abrir sus propios negocios^(7-8)^.

En Italia, por ejemplo, una investigación con estudiantes de enfermería evidenció que, a pesar del crecimiento de las oportunidades de trabajo autónomo, existe una falta de programas estructurados que preparen adecuadamente a los futuros enfermeros para carreras independientes^(9)^. En Brasil, la situación es similar: aunque los estudiantes de enfermería reconocen la importancia del emprendimiento, su abordaje durante la carrera es insuficiente, con pocas oportunidades para desarrollar competencias en esta área^(10)^.

Consonante con estos hallazgos, un estudio realizado en una universidad pública brasileña reveló que el 62,2% de los estudiantes de enfermería informaron que el emprendimiento es poco explorado durante la carrera^(11)^. Otra investigación, que analizó 130 cursos de enfermería de instituciones públicas de educación superior en Brasil, mostró que apenas el 10,8% incluía en la malla curricular y/o en el proyecto pedagógico una asignatura específica sobre emprendimiento; y, cuando estaba incluida, la mayoría (78,6%) ofrecía contenido exclusivamente teórico, sin el enfoque práctico necesario para el desarrollo de competencias^(12)^.

Otro estudio, realizado en la región Nordeste de Brasil, reveló que, entre las 412 Instituciones de Educación Superior (IES) que ofrecen la carrera de enfermería, apenas 37 disponibilizaron documentos curriculares completos, en los cuales se identificaron 17 asignaturas relacionadas con el emprendimiento y/o el empresariado. El análisis de los proyectos pedagógicos, planes de curso y programas identificó que la mayoría de esas asignaturas era teórica (94,1%), mientras que apenas el 5,9% incluía un enfoque práctico^(13)^. Estos datos refuerzan que la inclusión del tema en los currículos de enfermería aún es incipiente en Brasil.

Ante la carencia de formación emprendedora en este contexto, se carece de instrumentos para evaluar la preparación de los estudiantes de enfermería en relación con el emprendimiento de negocios, considerando las especificidades de esta área. Para cubrir esta necesidad y evaluar estrategias educativas, un grupo de investigadores en Italia desarrolló y validó el inventario *Livello di Preparazione Alla Carriera Libero Professionale* (LPACIP) con el objetivo de evaluar la preparación de los estudiantes de enfermería para actuar en carreras autónomas e independientes. El LPACIP, validado en Italia con estudiantes del último año de la carrera de enfermería, demostró buenas propiedades psicométricas, con índices adecuados de validez de constructo y consistencia interna^(14)^. Hasta el momento de la elaboración de este estudio, no se identificaron en la literatura adaptaciones transculturales ni validaciones del inventario en otros países.

Dada la relevancia del LPACIP y el potencial impacto en la educación emprendedora en la enfermería brasileña, que en los últimos años ha señalado una ampliación del interés por el emprendimiento de negocios, este estudio tuvo como objetivo traducir, adaptar y evaluar las propiedades psicométricas del inventario *Livello di Preparazione Alla Carriera Libero Professionale* para su uso entre estudiantes del último año de la carrera de enfermería en Brasil.

## Método

### Diseño del estudio

Se trata de un estudio metodológico, de corte transversal, desarrollado en dos fases. En la primera, se utilizó el proceso de adaptación transcultural para traducir y adaptar el LPACIP al contexto brasileño^(12)^. En la segunda, el inventario traducido y adaptado fue sometido a pruebas para evaluar sus propiedades psicométricas, con énfasis en la validez de constructo y la consistencia interna. El estudio fue redactado de acuerdo con la directriz STROBE (*Strengthening the Reporting of Observational Studies in Epidemiology*).

### Instrumento

El LPACIP está compuesto por dos subescalas^(14)^. La primera tiene como objetivo identificar los factores importantes para la fijación de precios de los servicios liberales en enfermería y contiene diez ítems distribuidos en dos dominios: *complessità dell’assistenza* (ítems del uno al siete) y *caratteristiche logistiche* (ítems del ocho al diez). Los ítems se evalúan en una escala ordinal de cinco puntos, en la que uno indica “nada importante” y cinco, “muy importante”. La segunda subescala aborda el conocimiento de las características relacionadas con el trabajo liberal en enfermería, compuesta por 24 ítems divididos en dos dominios: *conoscenza delle regole amministrative* (ítems del uno al 12) y *conoscenza delle questioni relative a pensioni e previdenza* (ítems del 13 al 24). Cada ítem se evalúa en una escala ordinal de cinco puntos, en la que uno indica “desinformado” y cinco, “totalmente informado”.

### Proceso de traducción y adaptación cultural

Siguiendo las directrices de adaptación transcultural^(15)^, el LPACIP fue traducido y adaptado al contexto brasileño mediante las siguientes etapas: traducción directa, síntesis de las traducciones, retrotraducción, revisión por comité de expertos, prueba piloto y aprobación de la versión adaptada por el autor del instrumento original.

En la traducción directa, dos traductores independientes, ambos brasileños y bilingües en italiano, tradujeron el instrumento original al portugués de Brasil, generando dos versiones (T1 y T2). El traductor uno tenía experiencia y conocimiento de los conceptos del instrumento, mientras que el traductor dos no poseía formación en el área de la salud y no fue informado sobre los conceptos. En la síntesis de las traducciones, T1 y T2 fueron cuidadosamente comparadas, y los traductores e investigadores desarrollaron la primera versión consensuada (T1-2). La versión T1-2 fue retrotraducida al italiano de forma independiente (RT1 y RT2) por dos traductores bilingües en portugués, que no poseían formación en el área de la salud y no estaban familiarizados con los conceptos del inventario.

El comité de expertos fue seleccionado por conveniencia, considerando la experiencia y disponibilidad de los miembros para contribuir con la adaptación transcultural del instrumento. El grupo estuvo compuesto por dos docentes de enfermería con experiencia en adaptación transcultural, una enfermera emprendedora con cinco años de actuación exclusiva en el área, un estudiante del último año de la carrera de enfermería, representando al público objetivo, y los cuatro traductores involucrados en el proyecto.

El contacto inicial se realizó por correo electrónico y, tras aceptar participar y firmar el Consentimiento Informado (CI) específico para esta fase, los expertos recibieron los ítems traducidos para compararlos con la versión original. El comité fue responsable por consolidar la versión prefinal del instrumento. La evaluación de la versión traducida consideró las equivalencias conceptual, cultural, semántica e idiomática. Para este análisis, se utilizó una escala ordinal de cuatro puntos, en la que uno correspondía a “no equivalente” y cuatro a “muy equivalente”, lo que permitió validar el contenido de cada ítem. Las puntuaciones atribuidas se utilizaron para calcular el Índice de Validez de Contenido por ítem (I-CVI) y por subescala (S-CVI), según se describe en la sección ‘Tratamiento y análisis de datos’. Tras esta etapa, se realizó una reunión en línea para discutir las discrepancias identificadas y aprobar la versión prefinal del inventario.

La prueba piloto se aplicó en línea entre febrero y marzo de 2024, mediante un cuestionario elaborado en *Google Forms*®, estructurado en dos partes. La primera consistía en preguntas sociodemográficas, y la segunda presentaba la versión prefinal del inventario aprobada por el comité de expertos. Además, la comprensión de los ítems fue evaluada mediante un espacio para comentarios al final de cada subescala, en el que los participantes respondieron a preguntas estructuradas sobre la claridad del lenguaje, dificultades de comprensión y si sus respuestas reflejaban con precisión lo que deseaban expresar.

Los criterios de inclusión fueron: (1) tener más de 18 años; (2) estar cursando el último año de la carrera; y (3) estar regularmente matriculado en una IES pública o privada en el curso de enfermería. La selección de los participantes se realizó de forma no probabilística y por adhesión voluntaria, sin restricción a instituciones predeterminadas. La divulgación se realizó mediante el *WhatsApp*® e *Instagram*®, tanto en cuentas personales como en grupos de investigación. Además, el equipo del macroproyecto colaboró en la distribución de la investigación para alcanzar participantes de diferentes regiones de Brasil. Se excluyeron los datos que no cumplían con los criterios.

El autor del instrumento original acompañó todas las etapas de la adaptación transcultural, revisando y aprobando las modificaciones realizadas, así como la versión final del instrumento.

### Participantes, recolección de datos y definición de la muestra

En la segunda fase, para evaluar las propiedades psicométricas, se aplicaron los mismos criterios de inclusión de la prueba piloto. Además, se analizaron únicamente los participantes que respondieron completamente a las subescalas, ya que las respuestas incompletas podrían comprometer la validez del instrumento. Aunque existen métodos para tratar datos ausentes en psicometría, la literatura indica la ausencia de un consenso sobre el enfoque ideal, y su aplicación práctica aún es limitada^(16)^. Por lo tanto, se excluyeron las respuestas incompletas y aquellas que no cumplían con los criterios establecidos.

Los participantes fueron definidos con el fin de asemejarse a la validación del inventario original realizada en Italia, que también incluyó a estudiantes de enfermería en el último año de la carrera^(14)^. La recolección de datos se llevó a cabo en línea entre abril y septiembre de 2024, mediante un cuestionario elaborado en *Microsoft Forms*®, cuya divulgación siguió las mismas estrategias que la prueba piloto. El cuestionario fue estructurado en dos partes: la primera recopiló datos sociodemográficos como género, edad, nacionalidad, región de residencia en Brasil, nombre de la IES y categoría administrativa (pública o privada). La segunda parte consistió en la versión final del inventario traducido y adaptado al contexto brasileño, denominado “Preparación para la Carrera Liberal”, Versión Brasil (VB), también conocido como LPACIP-VB tras el consenso de los especialistas.

El tamaño de muestra recomendado para el Análisis Factorial Confirmatorio (AFC) es de cinco a diez veces el número de ítems del instrumento^(17)^. La versión original del LPACIP estaba compuesta por 34 ítems, pero durante el proceso de adaptación transcultural, se excluyeron dos ítems, resultando en una versión final con 32 ítems en el LPACIP-VB. Así, el número estimado de participantes para esta fase varió de 160 a 320. Se obtuvieron 361 respuestas, de las cuales 33 fueron excluidas por no cumplir con los criterios de inclusión. Así, la muestra final estuvo compuesta por 328 participantes.

### Tratamiento y análisis de datos

Los datos fueron organizados en hojas de cálculo de *Microsoft Excel*® y analizados en el *software* estadístico Jasp (versión 0.19.1; Ámsterdam, Países Bajos). Se utilizaron estadísticas descriptivas para caracterizar la muestra. Las variables cuantitativas se analizaron mediante media, mediana, desviación estándar (DE), mínimo (Min.) y máximo (Max.), mientras que las variables nominales por frecuencias absolutas (n) y relativas (%).

Las puntuaciones asignadas por el comité de expertos se utilizaron para calcular el I-CVI y el S-CVI. El I-CVI representa la proporción de expertos que asignaron puntuaciones de tres o cuatro a cada ítem, mientras que el S-CVI corresponde al promedio del I-CVI de todos los ítems. De acuerdo con la literatura, valores de I-CVI ≥ 0,80 y S-CVI ≥ 0,90 se consideran adecuados para garantizar la validez de contenido^(18)^.

Para evaluar la validez de constructo, se realizó un AFC para cada subescala del inventario, utilizando el método de mínimos cuadrados ponderados diagonalmente (*Diagonally Weighted Least Squares* – DWLS) para la estimación de los parámetros. El AFC se condujo con una estructura de dos factores, la misma confirmada en la versión italiana^(14)^, con el fin de verificar su aplicabilidad en una muestra brasileña. Antes del AFC, se verificó la adecuación de la muestra mediante la prueba de Kaiser-Meyer-Olkin (KMO) y la prueba de esfericidad de Bartlett, adoptándose como criterio un KMO superior a 0,70 y significancia estadística (p ≤ 0,05) en la prueba de Bartlett^(19-20)^.

El AFC se realizó con el paquete *Latent Variable Analysis* (LAVAAN)^(21)^ y se evaluó la calidad del ajuste del modelo mediante los siguientes índices: razón chi-cuadrado/grados de libertad (χ²/gl), Índice de Ajuste Normalizado (*Normed Fit Index* – NFI), Índice de Ajuste Normalizado (*Normed Fit Index* – NFI), Índice de Ajuste Comparativo (*Comparative Fit Index* – CFI), Índice de Tucker-Lewis (TLI), Raíz del Error Cuadrático Medio de Aproximación (*Root Mean Square Error of Approximation* – RMSEA) y Raíz del Error Cuadrático Medio Residual Estandarizado (*Standardized Root Mean Square Residual* – SRMR). Además, se analizaron los coeficientes de correlación entre los factores latentes del modelo.

El ajuste del modelo se consideró adecuado cuando se cumplieron los siguientes criterios: χ²/gl inferior a 5,0^(19)^; índices NFI, CFI y TLI superiores a 0,90^(22)^; RMSEA inferior a 0,10 y SRMR menor que 0,08, lo que indica un ajuste aceptable^(22)^. Las cargas factoriales (λ) de los ítems se consideraron adecuadas cuando fueron superiores a 0,30^(19,23)^.

Adicionalmente, se calculó el coeficiente de determinación (R²), que mide la proporción de la varianza de la variable dependiente explicada por la variable independiente. El valor de R² varía entre cero y uno, siendo que valores más cercanos a uno indican un excelente ajuste del modelo. En este estudio, el R² se utilizó para evaluar la calidad del ajuste del modelo en relación con los datos observados^(24)^. La consistencia interna se evaluó mediante los coeficientes alfa de Cronbach (α) y omega de McDonald (ω) para cada factor y para la subescala general, considerando como referencia valores superiores a 0,70 en ambos tests^(25-26)^.

### Aspectos éticos

El estudio fue aprobado por el Comité de Ética en Investigación bajo el dictamen n.º 6.430.383. Antes del inicio de la investigación, se obtuvo la autorización del autor del instrumento original para traducir, adaptar y validar el inventario en el contexto brasileño. Todos los participantes fueron informados sobre los objetivos del estudio y participaron de forma voluntaria, firmando el CI.

## Resultados

### Primera fase: traducción y adaptación cultural

La validez de contenido del LPACIP-VB fue evaluada por ocho expertos. En el proceso de adaptación transcultural, la primera subescala mantuvo diez ítems, presentando una excelente validez de contenido, con I-CVI variando de 0,875 a 1,000 y S-CVI de 0,975. En la segunda subescala, la mayoría de los ítems obtuvo I-CVI superior a 0,875, excepto los ítems 13 y 14 del factor dos, cuyos valores fueron 0,250 y 0,375, respectivamente.

Estos ítems abordaban normativas específicas del sistema previsional italiano, sin aplicabilidad en Brasil (Ítem 13: Funciones del Consejo de Administración del Instituto Nacional de Previsión Social y Asistencia a la Profesión de Enfermería; Ítem 14: Funciones del Consejo Consultivo General del Instituto Nacional de Previsión Social y Asistencia a la Profesión de Enfermería). En la reunión final de consenso, tras la discusión del comité de expertos, se decidió excluir estos ítems para garantizar que el factor dos reflejara con precisión las normativas y prácticas previsionales brasileñas. Con esta exclusión, el S-CVI final fue de 0,965. Así, la segunda subescala, que en la versión italiana posee 24 ítems^(14)^, pasó a tener 22 en la versión brasileña. Las traducciones y la versión final del LPACIP-VB están disponibles en la plataforma internacional *Open Science Framework* (OSF.io), bajo el identificador n.º 10.17605/OSF.IO/2SXD8 (https://doi.org/10.17605/OSF.IO/2SXD8).

Como resultado de la exclusión de los ítems, el cálculo de la puntuación para el factor dos de la segunda subescala fue ajustado. Cada dominio de ambas subescalas debe ser calculado estandarizando las puntuaciones brutas en una única puntuación de cero a 100. Así, para la primera subescala, en el dominio “complejidad asistencial”, el cálculo es: [(suma de los ítems 1 a 7)/7]; para el dominio “características logísticas”, el cálculo es: [(suma de los ítems 8 a 10)/3]. Para la segunda subescala, el dominio “conocimiento de cuestiones administrativas” se calcula como: [(suma de los ítems 1 a 12)/12] y el dominio “conocimiento sobre cuestiones de beneficios y jubilación” como: [(suma de los ítems 13 a 22)/10]. Puntuaciones más altas indican mejor preparación de los estudiantes para carreras liberales en enfermería^(14)^.

Los 47 estudiantes que participaron en la prueba piloto tenían edades entre 20 y 59 años, con una media de 24,91 (mediana = 24,00; DE = 6,08), siendo la mayoría de sexo femenino (87,23%). La distribución geográfica fue la siguiente: 23,40% del Nordeste; 21,28% del Norte; 21,28% del Centro-Oeste; 21,28% del Sur y 12,77% del Sudeste. Todos los participantes afirmaron no haber encontrado dificultades en la comprensión de los ítems de las subescalas.

### Segunda fase: análisis factorial confirmatorio y consistencia interna

La Tabla 1 presenta las características de los participantes de la segunda fase del estudio (n = 328). La distribución geográfica abarcó las cinco regiones de Brasil, con mayor concentración en la región Norte (44,21%). En cuanto a la categoría administrativa de las IES, la mayoría de los estudiantes (62,80%) estaba matriculada en instituciones privadas. En total, participaron estudiantes del último año de la carrera de enfermería de 34 IES, siendo la mayoría (70,58%) clasificada como universidad (Tabla 1).

**Tabla 1-:** Características de los participantes de la segunda fase del estudio. Belém, PA, Brasil, 2024

**Estudiantes de enfermería (n=328)**	**n** * **(%)** ^†^
Edad (años)	Media 27,50; mediana 24,00; DP ^‡^ 7,58, mínimo 20, máximo 60
Género	
Femenino	277 (84,46%)
Masculino	50 (15,24%)
Otro	1 (0,30%)
Nacionalidad	
Brasileña	327 (99,70%)
Otra	1 (0,30%)
Región de Brasil	
Norte	145 (44,21%)
Nordeste	81 (24,70%)
Centro-Oeste	40 (12,19%)
Sudeste	22 (6,71%)
Sur	40 (12,19%)
Categoría administrativa de las IES ^§^	
Privada	206 (62,80%)
Pública	122 (37,20%)
Organización académica das IES ^§^ (n*=34)	
Universidad	24 (70,58%)
Centro Universitario	5 (14,71%)
Facultad	5 (14,71%)

*n = Frecuencia absoluta; ^†^% = Frecuencia porcentual; ^‡^DP = Desviación estándar; ^§^IES = Instituciones de Educación Superior

A continuación, se presentan los resultados del AFC y de la consistencia interna de las subescalas uno y dos del inventario.

### Primera subescala: factores para determinar los precios de las actividades

La adecuación de los datos para el AFC fue confirmada por la prueba KMO de 0,864 y por la prueba de Bartlett significativa (χ² = 1516,778, gl = 45, *p* < 0,001). Las cargas factoriales estandarizadas se presentan en la Tabla 2 y variaron de 0,596 (ítem tres) a 0,920 (ítem nueve), todas significativas (*p* < 0,001), lo que confirma que los ítems presentan buenas correlaciones con sus respectivos factores. La covarianza entre los factores latentes reveló una correlación de 0,757 entre el factor uno y el factor dos, con un Intervalo de Confianza (IC) del 95% que varió entre 0,686 y 0,828, Error Estándar (EE) de 0,036 y *p* < 0,001. El análisis del R² mostró valores entre 0,355 (ítem tres) y 0,847 (ítem nueve), indicando que una proporción significativa de la varianza de los ítems fue explicada por los factores (Tabla 2).

El modelo presentó buenos índices de ajuste: χ²/gl = 2,0 (χ² = 70,871, gl = 34, *p* < 0,001); NFI = 0,979; CFI = 0,989; TLI = 0,986; RMSEA = 0,058 (IC 90% = 0,039–0,076) y SRMR = 0,066. Estos resultados confirman que la estructura factorial fue validada (Tabla 2). La consistencia mostró valores de α de Cronbach de 0,765 y ω de McDonald de 0,760 para el factor uno, mientras que para el factor dos, los valores fueron α de 0,673 y ω de 0,686. En la subescala general, el α fue de 0,812 y el ω de 0,811, lo que evidencia una confiabilidad satisfactoria (Tabla 3).

**Tabla 2-:** Análisis factorial confirmatorio de la subescala: factores para determinar los precios de las actividades. Belém, PA, Brasil, 2024

Ítems	Cargas factoriales	**Error-estándar**	**R** ^2^ *	**P-valor**
Factor uno: complejidad asistencial				
1 Grado de complejidad asistencial del servicio	0,649	0,043	0,422	0,001
2 Mismo servicio prestado en varias atenciones	0,610	0,042	0,372	0,001
3 Varios servicios en una misma atención	0,596	0,048	0,355	0,001
4 Solicitudes de servicios consecutivos, distintos del primero, por el mismo cliente	0,639	0,044	0,408	0,001
5 Planificación de las intervenciones asistenciales	0,775	0,040	0,601	0,001
6 Carácter de urgencia del servicio	0,827	0,033	0,683	0,001
7 Suministro de equipos, materiales e insumos médico-hospitalarios de uso habitual por el profesional	0,668	0,054	0,447	0,001
Factor dos: características logísticas				
8 Distancia del domicilio del cliente	0,619	0,044	0,383	0,001
9 Servicio en los periodos de noche y madrugada	0,920	0,031	0,847	0,001
10 Servicio en días festivos	0,763	0,038	0,583	0,001
χ²/gl ^†^ = 2,0				
CFI ^‡^ = 0,989				
TLI ^§^ = 0,986				
NFI ^||^ = 0,979				
RMSEA ^¶^ = 0,058 (IC 90%** = 0,039–0,076)				
SRMR ^††^ = 0,066				

*R^2^ = Coeficiente de determinación; ^†^χ²/gl = Chi-cuadrado/grados de libertad; ^‡^CFI = *Comparative Fit Index*; ^§^TLI = Tucker-Lewis *Index*; ^||^NFI = *Normed Fit Index*; ^¶^RMSEA = *Root Mean Square Error of Approximation*; **IC = Intervalo de confianza del 90%; ^††^SRMR = *Standardized Root Mean Square Residual*

**Tabla 3-:** Análisis de la consistencia interna de la subescala: factores para la determinación de los precios de las actividades. Belém, PA, Brasil, 2024

**Factores**	Ítems	**IC 95%** *	
		**Alfa de Cronbach**	**Omega de McDonald**
Factor uno: complejidad asistencial	1, 2, 3, 4, 5, 6, 7	0,765 (0,724–0,802)	0,760 (0,720–0,800)
Factor dos: características logísticas	8, 9, 10	0,673 (0,605–0,731)	0,686 (0,629–0,742)
Total		0,812 (0,779–0,840)	0,811 (0,781–0,842)

*IC 95% = Intervalo de Confianza del 95%

### Segunda subescala: conocimiento sobre las características relacionadas con la carrera liberal

La adecuación de los datos para el AFC fue confirmada por la prueba KMO de 0,904 y por la prueba de Bartlett significativa (χ² = 11800,135, gl = 231, *p* < 0,001). Todas las cargas factoriales estandarizadas fueron significativas (*p* < 0,001), variando de 0,417 (ítem 16) a 0,976 (ítem 21), según se presenta en la Tabla 4. La covarianza entre los factores también fue satisfactoria, con un valor de 0,806 (EE: 0,022; IC 95%: 0,764–0,848; *p* < 0,001), reflejando una buena correlación entre el Factor uno y el Factor dos.

El R² varió de 0,174 (ítem 16) a 0,953 (ítem 21), evidenciando que una proporción significativa de la varianza de los ítems fue explicada por los factores (Tabla 4). El ajuste del modelo presentó valores adecuados: χ²/gl = 3,5 (χ² = 729,788, gl = 208, *p* < 0,001); NFI = 0,998; CFI = 0,999; TLI = 0,998; RMSEA = 0,088 (IC 90% = 0,081 a 0,095; *p* < 0,000) y SRMR = 0,053, confirmando la estructura factorial (Tabla 4). La consistencia interna también fue satisfactoria. Para el factor uno, el α de Cronbach fue de 0,941 y el ω de McDonald de 0,944. En el factor dos, el α fue de 0,960 y el ω de 0,964. En la subescala general, el α fue de 0,965 y el ω de 0,967, lo que evidencia una confiabilidad excelente (Tabla 5).

**Tabla 4-:** Análisis factorial confirmatorio de la subescala: conocimiento sobre las características relacionadas con la carrera liberal. Belém, PA, Brasil, 2024

Ítems	**Cargas factoriales**	**Error-estándar**	**R** ^2^ *	**P-valor**
Factor uno: conocimiento sobre cuestiones administrativas				
1 Remuneración justa	0,739	0,030	0,546	0,001
2 Publicidad en salud	0,777	0,026	0,603	0,001
3 Formas de actuación del profesional liberal (clínica, consultorio o empresa de enfermería)	0,449	0,045	0,201	0,001
4 Apertura de una clínica, consultorio o empresa de enfermería	0,776	0,024	0,603	0,001
5 Gestión de la documentación del cliente en domicilio y/o en la clínica, consultorio o empresa de enfermería	0,933	0,010	0,871	0,001
6 Gestión de la documentación administrativa de la clínica, consultorio o empresa de enfermería	0,940	0,011	0,883	0,001
7 Privacidad del paciente (Ley General de Protección de Datos Personales)	0,694	0,031	0,001
8 Obligaciones regulatorias sobre elaboración de presupuestos y contractualización	0,901	0,012	0,001
9 Diferentes tipos de regímenes tributarios (simple nacional, lucro presumido, lucro real, ...)	0,972	0,007	0,001
10 Diferentes tipos de facturas (factura electrónica, factura electrónica de servicio, factura ocasional, ...)	0,942	0,010	0,001
11 Seguro de responsabilidad civil y póliza de accidentes	0,958	0,009	0,001
12 Papel del Consejo de Enfermería en relación con el profesional liberal	0,808	0,019	0,653	0,001
Factor dos: conocimiento sobre cuestiones de beneficios y jubilación				
13 Funciones del Instituto Nacional do Seguro Social (INSS)	0,934	0,007	0,873	0,001
14 Tipos de contribución previsional del Instituto Nacional do Seguro Social (INSS) para profesionales liberales (Plan Normal y Plan Simplificado)	0,963	0,005	0,927	0,001
15 Diferencias entre contribuciones previsionales del Instituto Nacional do Seguro Social (INSS)	0,958	0,005	0,917	0,001
16 Diferencias entre contribuciones previsionales del Instituto Nacional do Seguro Social (INSS) para profesionales liberales (contribución con base en el salario mínimo o con base en la renta individual)	0,417	0,036	0,174	0,001
17 Composición del monto de la contribución	0,950	0,006	0,903	0,001
18 Beneficios previsionales concedidos por el Instituto Nacional do Seguro Social (INSS) (plan normal: jubilación por edad, invalidez y tiempo de contribución, subsidio por enfermedad, salario de maternidad, auxilio por reclusión, pensión por fallecimiento, certificado de tiempo de contribución)	0,933	0,007	0,871	0,001
19 Beneficios previsionales concedidos por el Instituto Nacional do Seguro Social (INSS) (plan simplificado: jubilación por edad, subsidio por enfermedad, pensión por fallecimiento, jubilación por invalidez, salario de maternidad, auxilio por reclusión)	0,754	0,024	0,568	0,001
20 Diferencias entre las contribuciones previsionales y la tributación fiscal	0,974	0,003	0,948	0,001
21 Deducibilidad de la contribución previsional	0,976	0,003	0,953	0,001
22 Regularidad de la contribución	0,957	0,005	0,915	0,001
χ²/gl ^†^ = 3,5				
CFI ^‡^ = 0,999				
TLI ^§^ = 0,998				
NFI ^||^ = 0,998				
RMSEA ^¶^ = 0,088 (IC 90%** = 0,081–0,095)				
SRMR ^††^ = 0,053				

*R^2^ = Coeficiente de determinación; ^†^χ²/gl = Chi-cuadrado / grados de libertad; ^‡^CFI = *Comparative Fit Index*; ^§^TLI = Tucker-Lewis *Index*; ^||^NFI = *Normed Fit Index*; ^¶^RMSEA = *Root Mean Square Error of Approximation*; **IC = Intervalo de Confianza del 90%; ^††^SRMR = *Standardized Root Mean Square Residual*

**Tabla 5-:** Análisis de la consistencia interna de la subescala: conocimiento sobre las características relacionadas con la carrera liberal. Belém, PA, Brasil, 2024

**Factores**	Ítems	**IC 95%** *	
		**Alfa de Cronbach**	**Omega de McDonald**
Factor uno: conocimiento sobre cuestiones administrativas	1, 2, 3, 4, 5, 6, 7, 8, 9, 10, 11, 12	0,941 (0,932–0,950)	0,944 (0,935–0,953)
Factor dos: conocimiento sobre cuestiones de beneficios y jubilación	13, 14, 15, 16, 17, 18, 19, 20, 21, 22	0,960 (0,954–0,966)	0,964 (0,959–0,970)
Total		0,965 (0,959–0,970)	0,967 (0,962–0,973)

*IC 95% = Intervalo de Confianza del 95%

## Discusión

Los resultados de este estudio indican que el LPACIP-VB es un instrumento válido y confiable para evaluar la preparación de estudiantes del último año de la carrera de enfermería para carreras liberales. Siendo el primero validado con este propósito en Brasil, el inventario mantuvo su estructura factorial original, presentando propiedades psicométricas sólidas que confirman su validez de constructo y consistencia interna.

En el proceso de adaptación transcultural, la primera subescala mantuvo diez ítems, mientras que la segunda, originalmente compuesta por 24 en la versión italiana^(14)^, pasó a tener 22 en la versión brasileña, debido a la exclusión de los ítems 13 y 14 del segundo factor, los cuales abordaban normativas previsionales italianas sin aplicabilidad en Brasil. La decisión fue tomada por el comité de expertos, y el S-CVI de la segunda subescala resultó en 0,965 tras la exclusión de dichos ítems. La eliminación de ítems no genera necesariamente una brecha en el instrumento; por el contrario, eliminar elementos culturalmente inapropiados puede mejorar su calidad, reflejando con mayor precisión la realidad del país. Esta adaptación permite que el instrumento capture mejor la experiencia local, lo que mitiga interpretaciones ambiguas que podrían comprometer la validez de los resultados^(27-29)^.

La traducción, adaptación y validación del LPACIP en Brasil fueron motivadas por la ausencia de instrumentos validados para evaluar la preparación de estudiantes de enfermería para carreras liberales. Hasta la elaboración de este estudio, el LPACIP es el único instrumento que mide específicamente el conocimiento sobre el emprendimiento de negocios en el área, destacándose ante la escasez de medidas confiables que identifiquen los elementos esenciales para la práctica autónoma. Aunque fue probado solo una vez en su estudio original^(14)^, esta investigación representa su segunda aplicación, permitiendo evaluar sus propiedades psicométricas en un nuevo contexto cultural. Su estructura teórica consolidada y la necesidad de un instrumento adaptado a la realidad brasileña justifican su validación y aplicación en el país.

Como se destacó en el estudio original^(14)^, se requieren más investigaciones para comprobar la validez de constructo del LPACIP y verificar si los elementos de preparación se asocian con el éxito en la práctica liberal de la enfermería. Estudios futuros deben considerar análisis longitudinales y aplicaciones en diferentes muestras para fortalecer las evidencias de validez y confiabilidad, ampliando su aplicabilidad en diversos contextos.

El AFC confirmó que ambas subescalas mantuvieron una estructura factorial de dos factores, consistente con la versión original italiana^(14)^. Las cargas factoriales de la primera subescala variaron de 0,596 a 0,920, mientras que en la segunda subescala oscilaron entre 0,417 y 0,976, todas por encima del valor mínimo recomendado de 0,30^(19,23)^, lo que refuerza la adecuación del modelo teórico propuesto. Los índices de ajuste son medidas descriptivas que evalúan cuán bien se ajusta un modelo de AFC a los datos^(30)^.

En este estudio, los índices de ajuste del LPACIP-VB fueron similares o superiores a los de la versión italiana. En la primera subescala, el χ²/gl fue mejor en Brasil (2,0 frente a 3,2 en Italia), al igual que el RMSEA, que fue menor (0,058 frente a 0,075 en Italia). Además, los índices incrementales CFI (0,989) y TLI (0,986) superaron los de la versión italiana (CFI = 0,936; TLI = 0,907). En la segunda subescala, la versión brasileña presentó un ajuste inferior a la italiana. El χ²/gl fue de 3,5 en Brasil y 2,3 en Italia, y el RMSEA fue mayor en Brasil (0,088 frente a 0,058). No obstante, los índices incrementales fueron elevados en ambas versiones, con un CFI de 0,999 en Brasil frente a 0,957 en Italia, y un TLI de 0,998 frente a 0,950, lo que indica una buena adecuación del modelo. El SRMR fue de 0,053 en Brasil y de 0,033 en Italia^(14)^. A pesar de estas diferencias, todos los índices se mantuvieron dentro o próximos a los parámetros establecidos^(19,22)^, lo que garantiza la validez del instrumento en el contexto brasileño.

El Análisis Factorial Exploratorio (AFE) se utiliza ampliamente en las etapas iniciales del desarrollo de instrumentos, ya que su propósito es explorar la estructura factorial subyacente de los datos sin imponer una estructura previa^(31)^, lo cual ya se había realizado en la versión original del inventario^(14)^. En este estudio, se optó por el Análisis Factorial Confirmatorio (AFC), por ser el método más adecuado para probar y confirmar si la estructura empírica observada refleja el constructo teórico de interés en la población analizada^(32)^. De este modo, el AFC se empleó en la versión brasileña para evaluar la aplicabilidad y adecuación de la estructura factorial previamente identificada en la versión italiana en un nuevo contexto cultural. Este enfoque contribuye a garantizar la validez y confiabilidad del instrumento en diferentes idiomas y escenarios^(32)^.

En cuanto a la consistencia interna, considerando que el coeficiente alfa de Cronbach (α) puede verse influenciado por el número de ítems de un factor^(33)^, este estudio adoptó un enfoque complementario al utilizado en el proceso de validación del inventario original^(14)^. Además del α, también se calculó el coeficiente ω de McDonald, que ofrece una estimación más restrictiva de la confiabilidad al basarse en el modelo factorial (enfoque restrictivo), asumiendo que los ítems pueden tener diferentes cargas factoriales y medir distintos aspectos del constructo^(34-35)^. Aunque el ω es generalmente menos sensible al número de ítems en comparación con el α, factores con pocos ítems pueden presentar valores más bajos de consistencia interna^(26,36)^.

La consistencia interna fue adecuada para la mayoría de los factores y para las subescalas generales, con valores de α y ω superiores a 0,70, lo que refuerza la validez de constructo del instrumento. No obstante, el factor dos de la subescala uno, que contiene solo tres ítems, presentó valores ligeramente inferiores, aunque aún cercanos al valor de referencia establecido, lo que puede deberse al reducido número de ítems en el factor. Aunque el coeficiente α es comúnmente utilizado en detrimento de otras medidas, todavía no existe un consenso claro en la literatura sobre su interpretación^(34)^. Aun así, los valores de α obtenidos en este estudio fueron similares a los de la versión original del inventario^(14)^.

Se considera que las implicaciones de nuestros resultados van más allá de la validación psicométrica del LPACIP-VB. Al utilizar este instrumento, docentes e investigadores tendrán la oportunidad de evaluar de forma estructurada el nivel de preparación de los estudiantes del último año de enfermería en Brasil para ejercer de forma liberal, un aspecto cada vez más relevante dada la expansión del alcance profesional de los enfermeros, más allá de los entornos tradicionales de trabajo^(37)^.

Además, puede servir como referencia para orientar el desarrollo de planes de estudio más alineados con el fortalecimiento de las habilidades generales requeridas para la práctica liberal en enfermería. Por ejemplo, si la evaluación revela puntuaciones bajas en áreas como la determinación de precios o el conocimiento sobre las características de la carrera liberal, se pueden implementar estrategias específicas para reforzar estas áreas según las dificultades identificadas.

Este estudio presenta algunas limitaciones que deben considerarse. En primer lugar, la recolección de datos transversal limita la capacidad de evaluar la estabilidad de cada escala a lo largo del tiempo. En segundo lugar, aunque la muestra fue obtenida en diferentes regiones de Brasil, puede no representar completamente la diversidad de los estudiantes de enfermería que cursan el último año. Además, los hallazgos reflejan las características de la muestra y el contexto de aplicación del instrumento. Cambios en el perfil de los estudiantes, especialmente en aspectos socioeconómicos y demográficos, así como diferentes métodos de recolección de datos, pueden impactar su validez y confiabilidad. Por lo tanto, se recomiendan nuevas investigaciones para evaluar su aplicabilidad en otros contextos y poblaciones. Finalmente, el uso de medidas autoadministradas puede haber introducido un posible sesgo de respuesta o efectos de deseabilidad social^(38)^.

A pesar de estas limitaciones, el estudio contribuye a la literatura sobre el emprendimiento en enfermería al proporcionar el primer instrumento validado para el contexto brasileño, capaz de medir la preparación de los estudiantes de enfermería del último año para la práctica liberal. Además, puede utilizarse en investigaciones futuras para explorar dicha preparación en diferentes regiones de Brasil, contribuyendo al avance del conocimiento científico sobre esta temática.

## Conclusión

El estudio tradujo, adaptó y confirmó la validez de constructo y la consistencia interna del LPACIP–VB para evaluar la preparación de los estudiantes de enfermería del último año de la carrera en Brasil para actuar en carreras autónomas e independientes. Se infiere, por lo tanto, que el instrumento es eficaz para que docentes e investigadores identifiquen lagunas en la enseñanza sobre emprendimiento en enfermería en este público, permitiendo el desarrollo de estrategias teóricas y prácticas más alineadas con las verdaderas dificultades de los estudiantes.

Además, el LPACIP-VB fomenta la discusión sobre fijación de precios de los servicios, cuestiones administrativas y previsionales, aspectos fundamentales para que los futuros enfermeros puedan ejercer de manera liberal y desarrollar negocios más estructurados en el área – un conocimiento aún poco explorado en la formación académica. Por último, se destaca que el uso del inventario es gratuito, pero requiere la autorización del autor correspondiente.
